# Impact of Lithium‐Free Borate Additives on the Cycle Life and Calendar Aging of Silicon‐Based Lithium‐Ion Batteries

**DOI:** 10.1002/smsc.202500479

**Published:** 2025-11-04

**Authors:** Defu Li, Amanda L. Musgrove, Xiuyu Jin, Harry M Meyer, Gabriel Muldoon, Gabriel M. Veith, Gao Liu

**Affiliations:** ^1^ Energy storage and Distributed Resources Division Lawrence Berkeley National Laboratory Berkeley 94720 CA USA; ^2^ Chemical Sciences Division Oak Ridge National Laboratory Oak Ridge 37830 TN USA

**Keywords:** borate additives, electrolyte additives, Li‐ion batteries, silicon anodes, solid electrolyte interphase layers

## Abstract

Silicon‐anode lithium‐ion batteries (LIBs) suffer from limited cycle life and poor calendar life, constraining their large‐scale commercialization. Integrating additives into electrolytes is a simple and cost‐effective strategy to improve these aspects. The effects of lithium‐free boron‐based additives on cycling and calendar performance of high‐loading Si‐anode LIBs remain largely unexplored. In this work, the influence of five Li‐free borate additives, each with distinct molecular structures and elemental compositions, is systematically investigated. All additives enhance cycle life to varying extents. Notably, the addition of 1 v/v% tri(2,2,2‐trifluoroethyl) borate to the baseline electrolyte nearly doubles the cycle life at 50% state of health. This enhancement is attributed to three key factors. Specifically, borate additives 1) improve electrochemical activity, 2) act as anion receptors that interact with [PF_6_]^−^ anions and carbonate solvents to reduce electrolyte decomposition, and 3) promote the formation of a stable and polymeric solid electrolyte interphase layer. Furthermore, these additives exhibited negligible impact in mitigating leakage current during a 180 h voltage‐hold calendar‐aging test, indicating their limited effect in calendar life. These findings provide insight into the role of Li‐free borate additives in improving cycle life while addressing the knowledge gap regarding their influence on calendar aging.

## Graphical abstract

Borate additive mechanisms for enhanced electrolyte stability and solid electrolyte interphase (SEI) formation: a) Additives act as anion receptors, mitigating [PF_6_]^−^ decomposition. b) Additives decompose, contributing to a boron‐, lithium‐, and oxygen‐rich SEI.

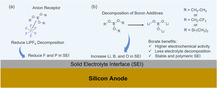

## 1 Introduction

Silicon (Si) is one of the most promising anode materials for high‐energy‐density lithium‐ion batteries (LIBs) due to its high theoretical specific capacity of ≈3700 mAh g^−1^.^[^ ^1^, ^2^, ^3^ ^]^ However, the large‐scale application of Si in LIBs is hindered by several major challenges, including limited cycle life and short calendar life.^[^ ^4^ ^]^ Over the past two decades, developments in electrolyte formulations,^[^ ^5^, ^6^ ^]^ electrode fabrication techniques,^[^ ^7^, ^8^ ^]^ and innovative polymer binders^[^ ^9^, ^10^ ^]^ have enabled Si‐based anodes to achieve a cycle life exceeding several hundred cycles.^[^ ^5^, ^11^ ^]^ However, it remains a challenge to achieve superior cycle life for LIBs with a high areal capacity (>4 mAh cm^−2^) of Si‐based anodes. In addition, none of these Si‐anode LIBs have exhibited a calendar life of more than 30 months.^[^ ^12^ ^]^ Therefore, addressing the short calendar life and limited cycle life of Si‐anode LIBs is crucial for advancing their practical applications.

Si particles offer high capacity for batteries but undergo substantial volume expansion (≈300%) and contraction during electrochemical lithiation and delithiation. These repetitive volume changes in Si particles can induce mechanical fracture, destabilize solid electrolyte interphase (SEI) layers, cause loss of electrical contact, and lead to progressive electrode degradation, factors that collectively contribute to rapid capacity decay and poor cycle life.^[^ ^8^, ^13^, ^14^ ^]^ The use of electrolyte additives has emerged as a simple and cost‐effective method to improve the cycling performance of LIBs and lithium metal batteries (LMBs).^[^ ^15^, ^16^, ^17^, ^18^, ^19^, ^20^, ^21^ ^]^ Electrolyte additives such as fluoroethylene carbonate (FEC) and vinylene carbonate (VC) are well established for their ability to promote the formation of stable and robust SEI layers on silicon anodes, thereby improving electrochemical performance and capacity retention.^[^ ^19^, ^20^, ^21^ ^]^ These additives facilitate the development of organic‐rich, polymeric SEI structures with enhanced mechanical elasticity, enabling them to more effectively accommodate the substantial volume changes of Si particles during cycling.^[^ ^19^, ^20^, ^21^, ^22^ ^]^

**Figure 1:**
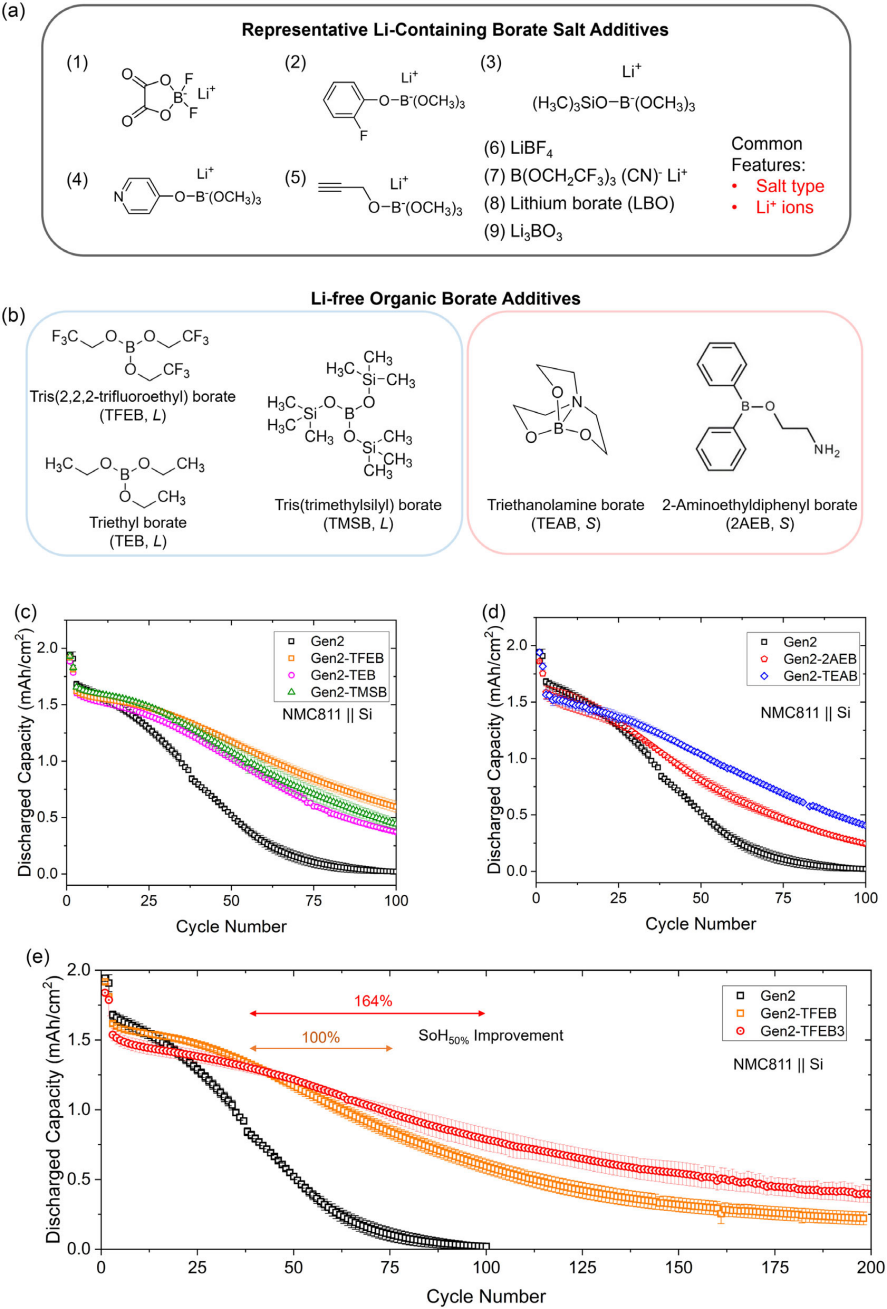
a) Representative examples of commonly used Li‐containing borate salts. b) Li‐free borate additives with varying molecular structures and chemical compositions, with “*L*” and “*S*” indicating their physical states at room temperature, corresponding to liquid and solid, respectively. c,d) Cycling performance of baseline Gen2 electrolytes containing (c) TFEB, TEB, and TMSB additives and (d) 2AEB and TEAB additives. e) Cycling performance at different concentrations of TFEB additive. The first and second cycles were conducted at C/25 and C/10 rates, respectively, with subsequent cycles at a C/3 rate. All abbreviations are defined in Table 1. Sample size: *n* ≥ 3.

Existing Li‐containing borate (Li‐borate) salt additives, which contain both lithium and boron, have been extensively investigated for use in cathodes and graphite anodes of LIBs, as well as lithium metal anodes in LMBs. As shown in **Figure** **1**a, representative examples include lithium difluoro(oxalato)borate (LiDFOB),^[^ ^23^, ^24^, ^25^, ^26^ ^]^ lithium cyano tris(2,2,2‐trifluoroethyl)borate,^[^ ^27^ ^]^ lithium alkyl trimethyl borates,^[^ ^28^ ^]^ lithium aryl trimethyl borates,^[^ ^28^ ^]^ and lithium borate (LBO).^[^ ^29^ ^]^ For instance, lithium bis(oxalato) borate (LiBOB), one of the most common additives, is well known for forming boron‐containing and robust cathode electrolyte interphases (CEIs) and SEIs, which suppress side reactions at electrode surfaces and improve electrochemical cycling performance.^[^ ^25^ ^]^ Innovative additives, such as lithium cyano tris(2,2,2‐trifluoroethyl) borate, have been synthesized and shown to generate a thin LiF‐rich SEI with low boron (B) and nitrogen (N) content, effectively suppressing lithium dendrite formation and significantly enhancing the cycle life of LMBs.^[^ ^27^ ^]^ Owing to the electron‐deficient nature of boron atoms within borate additives, they can interact with anions, such as [ClO_4_]^−^ and [PF_6_]^−^, to facilitate the dissociation of lithium salts.^[^ ^27^, ^30^ ^]^ In addition, the interaction between boron atoms and [PF_6_]^−^ anions mitigates the decomposition of [PF_6_]^−^ anions into undesirable byproducts.^[^ ^31^ ^]^ Overall, the interfacial chemistry, electrochemical behavior, and passivation mechanisms of these Li‐borate salts have been well studied.

In contrast, Li‐free organic borate additives remain far less studied, and their practical applications and fundamental mechanisms are not yet well understood. The primary distinction from Li‐borate salts lies in the absence of lithium within their molecular structures, meaning they do not contribute Li^+^ to the overall Li^+^ inventory of cells. Although several Li‐free borate additives have been reported, most studies have focused on their effects in cathodes. For example, trimethyl borate has been reported to form a protective interphase on cathode surfaces, thereby mitigating electrolyte decomposition, preventing structural degradation, and improving stability during high‐voltage operation.^[^ ^31^, ^32^ ^]^ However, systematic investigations into their role in Si anodes, particularly in the context of calendar aging, are largely lacking.

In this study, five Li‐free borate additives, each possessing distinct molecular structures and chemical compositions, were introduced into a carbonate electrolyte to explore their influence on the cycle life and calendar life of Si‐based LIBs. These five borate additives include tri(2,2,2‐trifluoroethyl) borate (TFEB), triethyl borate (TEB), tri(trimethylsilyl) borate (TMSB), triethanolamine borate (TEAB), and 2‐aminoethyldiphenyl borate (2AEB). All these borate additives contain a boron atom and possess boron–oxygen bonds. To evaluate their impact on cycle life, a high‐Si‐loading anode with 80 wt% Si, 10 wt% P84 polyimide (PI), and 10 wt% carbon was intentionally chosen to expedite the assessment of the borate additives, due to the short cycle life caused by the high Si content. Although these five borate additives differ in molecular structures and chemical compositions, they all noticeably improved the electrochemical activity and cycle life of high‐loading Si‐anode LIBs to varying extents. These observations suggest that the molecular structures and chemical compositions of borate additives significantly influence electrochemical performance. Moreover, these borate additives exhibit a common feature that promotes the formation of lithium‐, oxygen‐, and boron‐rich SEI layers.

## 2 Results and Discussion

Five commercially available Li‐free borate additives with distinct molecular structures and elemental compositions are systematically investigated to assess their influence on the electrochemical cycling and calendar aging of Si‐anode LIBs, as well as the underlying mechanisms. As shown in Figure 1b, TFEB, TEB, and TMSB are liquids at room temperature, whereas TEAB and 2AEB are solids. To accommodate these different physical states, additive contents were quantified using volume percentage (v/v%) for liquids and weight percentage (wt%) for solids.

### 2.1 Extended Cycle Life

Negative electrodes with a high loading of 80 wt% Si particles (300–500 nm in diameter) were intentionally chosen to expedite the evaluation of borate additives, as their inherently short cycle life facilitates rapid performance assessment. The NMC811 positive electrodes and Si‐based negative electrodes featured an areal capacity of 2.59 and 2.0 mAh cm^−2^, respectively, with a negative‐to‐positive (N/P) ratio of 0.77. In Figure 1c, the NMC811||Si full‐cell batteries using only Gen2 electrolytes experienced rapid capacity decay, achieving 50% state‐of‐health (SoH_50%_) after 39 cycles and losing all capacity before reaching 100 cycles. The rapid capacity fading is primarily attributed to the severe volume expansion and contraction of Si particles during lithiation and delithiation processes, structural degradation, and the depletion of bulk electrolyte and Li^+^ ion inventory in the cathode.

Under identical testing conditions, a low concentration (1 v/v% or 1 wt%) of any of these five Li‐free borate additives significantly enhanced battery cycling performance regardless of their liquid or solid state at room temperature. Among the liquid‐state additives, Gen2‐TFEB improved the SoH_50%_ by nearly 100%, while Gen2‐TMSB and Gen2‐TEB achieved improvements of 69% and 62%, respectively (Figure 1c, **Table** **1**). Among the solid‐state additives, Gen2‐TEAB and Gen2‐2AEB delivered enhancements of 74% and 31%, respectively (Figure 1d). These variations suggest that molecular structure and elemental composition strongly influence electrolyte properties and SEI formation, thereby affecting cycle life. For instance, TFEB and TEB share the same molecular structure, differing only in that TFEB contains fluorine atoms whereas TEB contains hydrogen atoms (Figure 1b). TFEB yielded higher capacity retention and greater cycle life stability than TEB, highlighting the beneficial role of fluorine in cycling performance. In contrast, TMSB, which contains a bulky Si‐(CH_3_)_3_ terminal group, showed negligible influence from its Si component on cycle life.

**Table 1:** Electrolyte formulation, cycle information (SoH_50%_), and Coulombic efficiency at first cycle.

Electrolyte composition	Abbreviation	Cycle number (SoH_50%_)	Improvement (SoH_50%_,%)	CE (1st cycle, %)
1.2 M LiPF6 in EC:EMC	Gen2	39	NA	69.0 ± 0.8
Gen2 + 1 v/v% TFEB	Gen2‐TFEB	78	100%	68.5 ± 0.2
Gen2 + 1 v/v% TEB	Gen2‐TEB	63	62%	68.2 ± 0.5
Gen2 + 1 v/v% TMSB	Gen2‐TMSB	66	69%	69.3 ± 0.6
Gen2 + 1 wt% TEAB	Gen2‐TEAB	68	74%	67.9 ± 0.5
Gen2 + 1 wt% 2AEB	Gen2‐2AEB	51	31%	68.9 ± 1.5
Gen2 + 3 v/v% TFEB	Gen2‐TFEB3	103	164%	64.6 ± 0.7

In the first cycle, SEI formation resulted in Coulombic efficiencies (CEs) of 67–69% for all cells using Gen2 electrolytes or their borate‐containing derivatives. The addition of any of the five borate additives produced CEs comparable to those of the baseline electrolyte, indicating that the first‐cycle CE is primarily governed by the baseline electrolyte composition, with low concentrations (1 v/v% or 1 wt%) of borate additives exerting only a limited influence.

Among the borate additives evaluated, TFEB delivered the greatest improvement in cycle life. Increasing the TFEB content from 1 v/v% to 3 v/v% extended the cycle life at SoH_50%_ from 78 to 103 cycles (Figure 1e, Table 1). However, the higher loading also led to a reduction in areal capacity during the first 40 cycles and a decrease in the first‐cycle CE. As TFEB contains no lithium within its molecular structure, the reduction or decomposition of a greater amount of additive or solvent consumes additional Li^+^ ions from the existing inventory during SEI formation. These Li‐free additives facilitate the formation of a more organic and polymeric SEI, which enhances its stability and prolongs cycle life. This explains why a higher TFEB content reduces the first cycle CE and initial areal capacity yet improves cycling performance. These findings suggest that the Li‐free TFEB encounters a tradeoff between maximizing areal capacity and achieving superior capacity retention, particularly in the early stages of cycling.

### 2.2 Improved Electrochemical Activity

**Figure 2:**
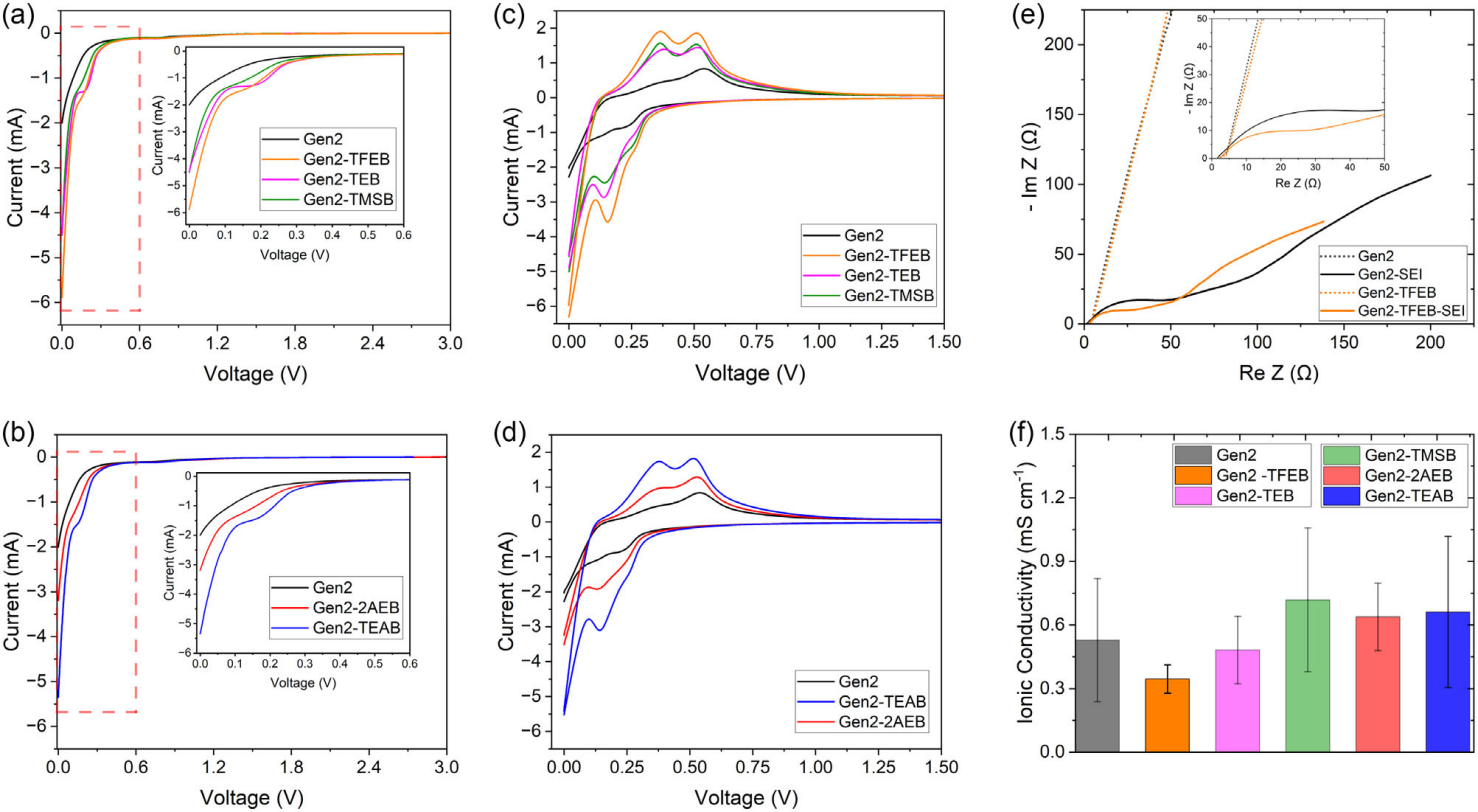
Electrochemical behaviors. a) LSV and b) CV of Gen2 electrolytes containing TFEB, TEB, and TMSB additives. c) LSV and d) CV of Gen2 electrolytes containing 2AEB and TEAB additives. e) EIS spectroscopy of NMC811||Si full cells with Gen2 or Gen2‐TFEB electrolytes, measured before and after SEI formation. f) Ionic conductivity.

Electrochemical activity governs the redox processes at the electrodes and is a key factor in determining battery capacity and cycle life. As shown in **Figure** **2**a,b, linear sweep voltammetry (LSV) was carried out from the open circuit potential (OCV, ≈3 V) to 0.01 V to evaluate the effect of borate additives in Gen2 carbonate‐based electrolytes. Subsequently, cyclic voltammetry (CV) scans were recorded from 0 V to 3 V. Representative first‐cycle CV profiles are presented in Figure 2c,d, with multicycle CV results provided in Figure S1 and S2, Supporting Information.

The LSV curves reveal that the baseline Gen2 electrolyte initiates lithiation at ≈0.35 V, whereas the borate‐containing electrolytes exhibit a slightly higher onset potential of ≈0.4 V (Figure 2a,b). Below 0.4 V, cells with borate additives deliver markedly higher current densities, highlighting their role in enhancing electrochemical activity. Silicon lithiation typically initiates at ≈0.4 V, with a characteristic cathodic peak appearing at 0.18–0.20 V during the first cycle and progressively shifting to lower potentials in subsequent cycles due to increasing passivation and interfacial resistance. As shown in Figure 2a,c and S1, Supporting Information, this peak was barely observable for the Gen2 electrolytes in the LSV curve and first CV cycle, but appeared at 0.1 V in the second cycle, and 0.06 V in the third. In contrast, the Gen2‐TFEB, Gen2‐TEB, and Gen2‐TEAB electrolytes exhibited this peak in both the initial LSV and first CV cycle (Figure 2a–d), followed by a similar shift to lower voltages in later cycles (Figure S1 and S2, Supporting Information). The progressive voltage shift reflects the accumulation of passivation layers and increased resistance at the Si electrode. Borate additives induced a strong cathodic peak at 0.15 V and an anodic peak at 0.36 V, both associated with the formation of amorphous Li_x_Si (a‐Li_*x*_Si, 0 ≤ *x* ≤ 3.75).^[^ ^33^, ^34^ ^]^ The adjacent satellite peaks correspond to the formation of crystalline Li_15_Si_4_ (c‐Li_15_Si_4_).^[^ ^33^, ^34^, ^35^ ^]^ The cathodic peaks observed at lower voltages reflect the partial transformation of a‐Li_*x*_Si to c‐Li_15_Si_4_ and the formation of c‐Li_15_Si_4_.^[^ ^34^, ^35^ ^]^ In the anodic scans, peaks at 0.36 and 0.52 V correspond to delithiation from a‐Li_*x*_Si and c‐Li_15_Si_4_ alloy, respectively. In the subsequent cycles, both cathodic and anodic peak intensities of a‐Li_*x*_Si increased, while the borate additives also promoted higher current densities near 0 V, corresponding to c‐Li_15_Si_4_ formation. These results indicate that the borate additives facilitate the formation of both a‐Li_*x*_Si and c‐Li_15_Si_4_ at the same scan rate, thereby enhancing lithiation and delithiation kinetics through improved electrochemical activity.

The cycling performance of these electrolytes strongly correlated with their electrochemical activity. Gen2‐TFEB showed the highest electrochemical activity, corresponding to the best cycle life. Gen2‐TMSB and Gen2‐TEB displayed comparable electrochemical activity and, consequently, similar cycle life outcomes. In contrast, Gen2 demonstrated the lowest electrochemical activity, yielding the poorest cycle life. This trend was also observed for Gen2‐TEAB and Gen2‐2AEB, confirming that borate additives improve electrochemical activity and, in turn, extend battery cycle life.

Gen2‐TFEB resulted in the longest cycle life among all tested electrolytes. Therefore, electrochemical impedance spectroscopy (EIS) experiments were carried out for both Gen2‐TFEB and the baseline Gen2 electrolyte, both before and after SEI formation, as illustrated in Figure 2e. Prior to formation, NMC811||Si full cells with either electrolyte exhibited comparable impedance. After one SEI formation cycle at a C/20 rate, Gen2‐TFEB exhibited a significantly lower interface resistance (26.6 Ω) than Gen2 (39.0 Ω) (Figure 2e and S3, Supporting Information), indicating that TFEB facilitates the formation of a low‐impedance SEI, thereby enhancing ion transport, improving Coulombic efficiency, and extending cycle life. In addition, the introduction of these borate additives resulted in negligible changes in bulk ionic conductivity (Figure 2f and S4, Supporting Information), suggesting that ionic conductivity was not the primary factor governing the observed enhancement in cycling performance.

### 2.3 Boron‐Based Robust SEI Layer

**Figure 3:**
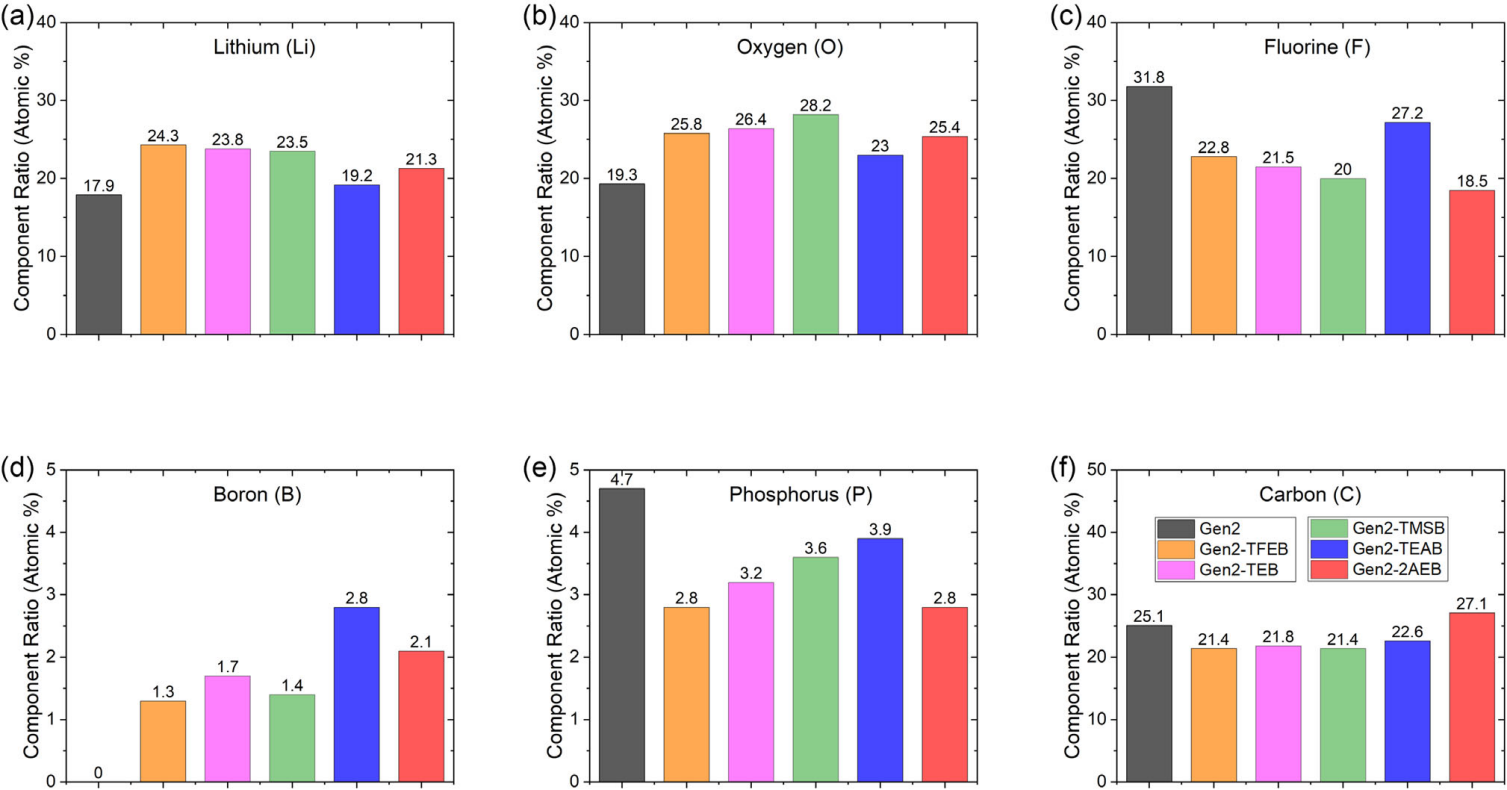
SEI surface composition ratio of a) lithium, b) oxygen, c) fluorine, d) boron, e) phosphorus, and f) silicon.

After three SEI formation cycles, the Si composite electrodes were rinsed with dimethyl carbonate (DMC) to remove residual electrolyte salts and soluble species, thereby obtaining the insoluble SEI layer. The surface elemental composition and chemical bonding states of this insoluble SEI were subsequently analyzed by X‐ray photoelectron spectroscopy (XPS) (**Figure** **3**; S5 and S6, Supporting Information).

In comparison to Gen2, the SEI layers formed with TFEB, TEB, and TMSB additives exhibited a 31–35% increase in lithium (Li) content and a 34–36% increase in oxygen (O) content (Figure 3a,b). However, the fluorine (F), phosphorus (P), and carbon (C) contents decreased by ≈30%, ≈25–40%, and ≈10%, respectively (Figure 3c,e,f), indicating that borate additives induce substantial changes in the organic and inorganic constituents of the SEI. While SEI layers from Gen2 contained no detectable boron (0%), those formed with borate additives contained 1–2 wt% boron (B) (Figure 3d), confirming boron's participation in SEI formation. The P content must originate from LiPF_6_ decomposition, whereas the B content arises from borate additive breakdown. Although P content decreased with borate addition, B content increased, and the combined P + B content was comparable to or greater than that of the Gen2 control. These results indicate that borate additives decompose in place of PF_6_^−^ anions, contributing to SEI formation. The F content, derived from PF_6_^−^ decomposition, was reduced in the presence of borate additives, indicating suppressed PF_6_^−^ breakdown. Under these conditions, the SEI is anticipated to possess a more organic or polymeric character, enhancing its stability and flexibility and thereby improving its ability to accommodate the volumetric expansion and contraction of Si particles during cycling. Furthermore, borate additives supplied additional C and O to the SEI, compensating for the loss of organic species during rinsing. As a result, the O content increased, whereas the C content remained nearly unchanged.

As shown in Figure 1b, TFEB, TEB, and TMSB each feature a central boron atom and share a similar star‐shaped, three‐branched molecular structure, but differ in their terminal group chemistry. These differences in terminal groups had a limited influence on SEI surface composition. For instance, although each TFEB molecule contains nine additional F atoms, its fluorine levels on the SEI surface were comparable to those of TEB and TMSB. In addition, while TMSB contains three Si atoms and six additional carbon atoms, the silicon and carbon levels detected on its SEI surface were similar to those of TFEB and TEB. These findings suggest that the terminal groups of these three borate additives had limited influence on SEI formation. Therefore, it is reasonable to hypothesize that they undergo decomposition, potentially yielding tri‐lithium borate (Li_3_BO_3_) as a byproduct,^[^ ^36^ ^]^ which contributes to SEI growth and enriches Li, B, and O content. TFEB, TEB, and TMSB exhibited similar trends in compositional changes within the SEI, whereas TEAB and 2AEB followed different patterns. This difference likely arises from molecular structure effects, as both TEAB and 2AEB contain nitrogen, leading to a marked increase in nitrogen content in the SEI. Collectively, these observations indicate that both the molecular structure and elemental composition of borate additives influence SEI chemistry and formation.

**Figure 4:**
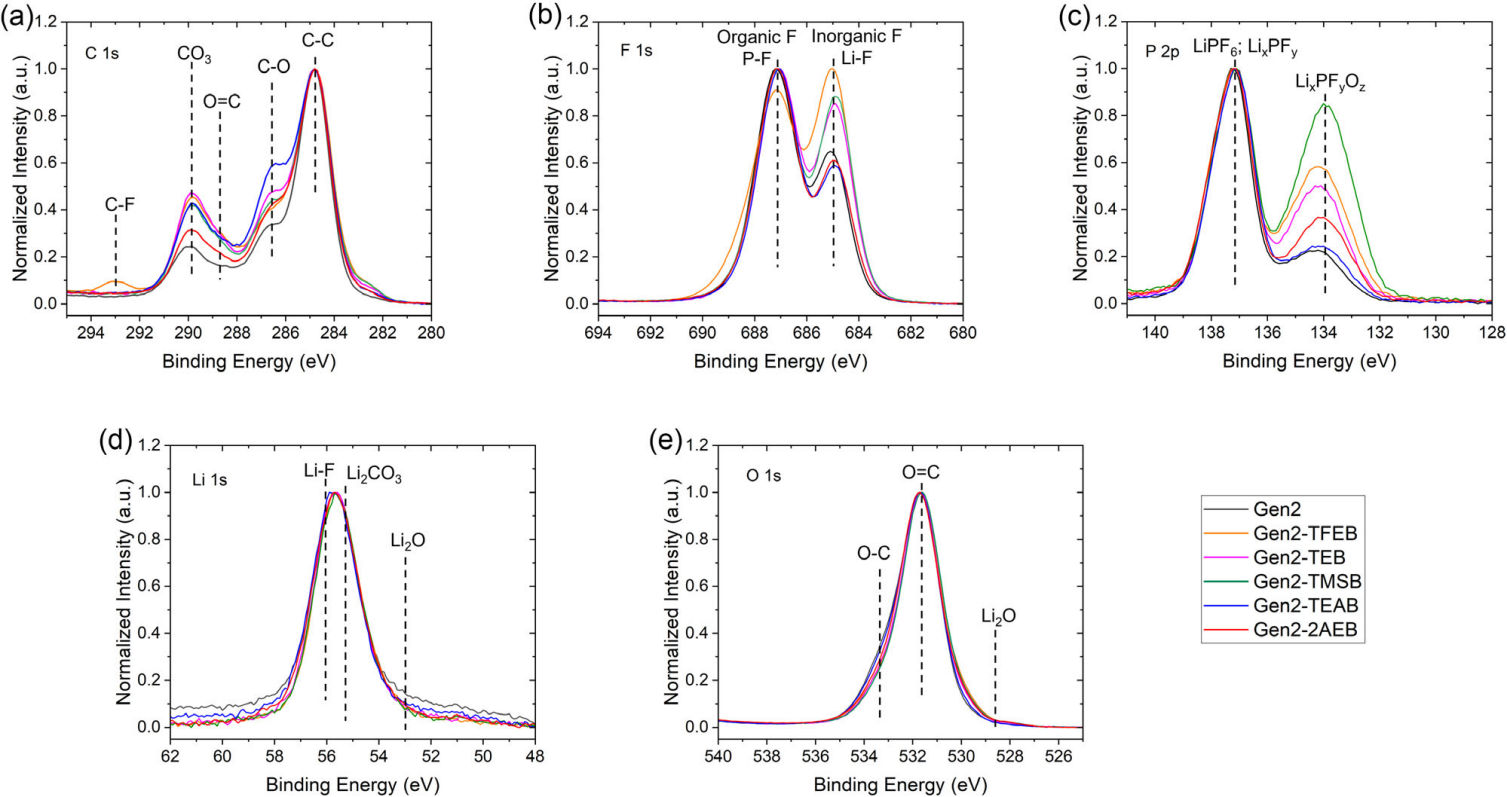
Normalized XPS spectra for the washed SEI surface of the Si composite electrodes. a) Carbon, b) fluorine, c) phosphorus, d) lithium, and e) oxygen.

In the carbon 1s spectra (**Figure** **4**a; S5 and S7, Supporting Information), the Si anode with Gen2 exhibited three major peaks at ≈284.8, 286.2, and 288–291 eV, corresponding to C—C, C—O, and C=O, respectively.^[^ ^31^, ^37^ ^]^ All borate additives exhibited a similar C 1s pattern, suggesting that these borate additives had negligible influence on the carbon species within the SEI layers. Organic solvents, carbon additives, polymer binders, borate additives, and SEI layers collectively contributed to the C 1s peaks, thereby complicating precise peak assignments. The C—C bond peak at ≈284.8 eV primarily originates from electrically conductive carbon black additives. The C=O bond peaks at 288–291 eV are attributed to lithium carbonate (LiCO_3_) and lithium alkyl carbonates (ROCO_2_Li).^[^ ^37^ ^]^ The C—O peak at ≈286.2 eV signified the bonding between a carbon atom and an oxygen atom, which is characteristic of lithium alkoxides, lithium alkyl carbonates, and PEO oligomers.^[^ ^37^ ^]^ In the O 1s spectra, the characteristic C—O peak was observed at ≈532.9 eV, providing additional evidence of the presence of these chemical compounds (Figure 4e). In addition, the C—F peak at ≈293 eV was associated with TFEB. The CO_3_ peak (≈289.9 eV) is primarily attributed to solvent decomposition, with higher peak intensities indicating more solvent decomposition. The incorporation of borate additives altered the SEI chemistry, favoring the formation of organic components over inorganic components. Notably, the borate additives that confer superior cycle life exhibit elevated CO_3_ peak intensities.

The F 1s spectra displayed two peaks at ≈687.2 and 685 eV (Figure 4b and S8, Supporting Information). The peak at ≈685 eV referred to LiF,^[^ ^38^, ^39^ ^]^ which formed from reaction byproducts or the decomposition of LiPF_6_ and electrolyte solvents. The broad peak, centered at 687.2 eV ranging from 686 eV to 690 eV, was primarily attributed to the LiPF_6_ (688 eV),^[^ ^39^ ^]^ Li_*x*_PO_*y*_F_*z*_ (≈687 eV),^[^ ^37^ ^]^ and POF_*x*_. In the Gen2‐TFEB sample, the F 1s spectrum exhibited higher intensity near 689–690 eV, attributed to the C—F bond of TFEB at 689.9 eV.^[^ ^38^ ^]^ This corresponds to the C—F peak observed in the C 1s spectra. This broad F 1s peak possibly included the B—F bond at 687.2 eV,^[^ ^31^ ^]^ which was a decomposition product of the borate additives. The electrolytes, with or without the borate additives, all featured a peak located exactly at 687.2 eV and shared a similar peak pattern. Thus, it was challenging to determine the presence of the B—F or quantify its proportion. The addition of TFEB, TEB, and TMSB into the Gen2 electrolytes significantly enhanced cycle life. In the baseline Gen2 electrolyte, the peak intensity at 687.2 eV exceeded that at ≈685 eV. However, the introduction of these additives reversed this trend. All three additives displayed a similar pattern, wherein the peak intensity at 687.2 eV was lower than that at ≈685 eV. This observation is attributed to the formation of SEI layers with increased organic species content, which are more readily removed than inorganic compounds during the washing process. Consequently, a relatively higher peak intensity corresponding to inorganic F and LiF (≈685 eV) was detected. However, it is important to note that the addition of borate additives such as TFEB, TEB, TMSB, and 2AEB led to a reduction of F content by nearly 30–40%, which facilitated the formation of SEI layers with enhanced organic and polymeric composition. The SEI layers featured less P and F content owing to the borate molecules acting as anion receptors, which interact with PF_6_^−^ anions and inhibit their decomposition. Among the borate additives evaluated, the relative peak intensity at 685 eV shows a strong correlation with both cycle life and electrochemical activity. Specifically, when the reduced F content is comparable, TFEB exhibits the highest LiF peak intensity, followed by TMSB, TEB, and 2AEB. This trend is consistent with the observed cycle life, where TFEB delivers the longest cycle life, followed in order by TMSB, TEB, and 2AEB. The electrochemical stability and mechanical robustness of LiF can possibly increase the structural stability and integrity of SEI layer, thereby enhancing cycle life. These findings suggest that both organic species and LiF compounds within the SEI layers play a critical role in determining cycle life.

In addition, the P 2p spectra exhibited two distinct peaks at ≈137 eV and ≈134 eV (Figure 4c and S9, Supporting Information). The peak at ≈137 eV is attributed to contributions from both the residual LiPF_6_ electrolyte component and its decomposed product Li_*x*_PF_*y*_. The peak at ≈134 eV is associated with Li_*x*_PF_*y*_O_*z*_, which is considered a byproduct of the LiPF_6_ electrolyte decomposition process.^[^ ^37^ ^]^ Notably, TFEB, TEB, and TMSB were the top three contributors to cycling performance, with a relatively higher amount on their SEI surface compared to the baseline electrolyte, TEAB, and 2AEB. Furthermore, in the Li 1s spectra, one broad peak ranging from 53 to 58 eV was observed. The peaks of Li_2_O, Li_2_CO_3_, and LiF typically appeared at 53, 55.3, and 56 eV (Figure 4d), respectively.^[^ ^27^ ^]^ By using the Gaussian method to fit the Li 1s spectra, their distribution was described in Figure S10, Supporting Information. The Li 1s and O 1s spectra results suggested a low amount of Li_2_O on the SEI layer surface, as indicated by the Li—O characteristic peak appearing at ≈528 eV in the O 1s spectra (Figure 4e and S11, Supporting Information) and ≈53 eV in the Li 1s spectra.^[^ ^37^ ^]^ The SEI layer surface, when exposed to fluoride‐containing electrolytes, exhibits the thermodynamic instability of Li_2_O, resulting in its low concentration on the SEI surface.^[^ ^37^, ^40^ ^]^ In comparison, LiF and Li_2_CO_3_ were identified as the major contributors to the Li 1s peak (Figure S10, Supporting Information).

These XPS results indicate that borate additives preferentially decompose in place of LiPF_6_, thereby mitigating LiPF_6_ decomposition. In addition, they enhance the decomposition of organic solvents, leading to the formation of a polymeric, organic‐rich SEI layer.

### 2.4 Surface and Cross‐Sectional Morphology

**Figure 5:**
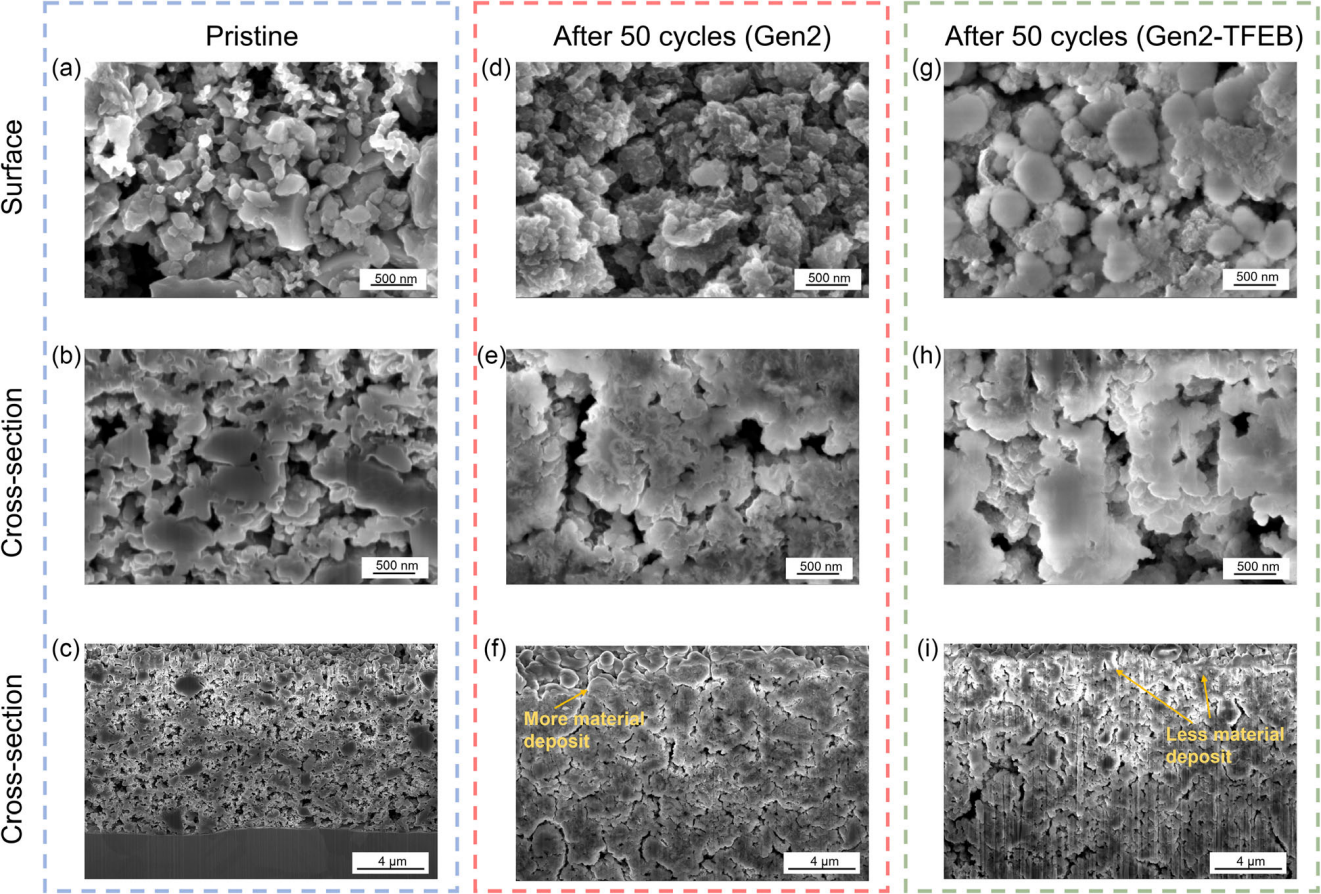
Surface and cross‐sectional morphology evolution of Si‐based electrodes. Surface and cross‐sectional SEM images of Si electrodes in a–c) pristine state, d–f) after 50 cycles with Gen2, and g–i) after 50 cycles with Gen2‐TFEB.

Material deposition on the electrodes resulting from electrolyte decomposition, coupled with substantial volume variations of Si particles during lithiation and delithiation, can impact the structural integrity of the electrode material and consequently affect cycling performance. As shown in **Figure** **5**a and S12, Supporting Information, the pristine Si electrodes feature a porous surface with discrete, irregularly shaped Si particles. In addition, these Si electrodes possess a porous cross‐sectional morphology that facilitates the penetration of electrolytes and enhances mass transport (Figure 5b,c and S13, Supporting Information), allowing access to the deeper Si particles at the bottom.

After 50 cycles using the Gen2 electrolyte, the electrodes exhibited large Si aggregates and clusters, significant cracks, and thick material deposits (Figure 5d–f; S14 and S15, Supporting Information). The cyclic volume expansion and contraction of Si particles during cycling induced repeated cracking and reformation of the SEI layers, progressively depleting electrolytes and resulting in accumulated material deposits on both the Si particles and the electrode surface. These deposits bridged adjacent Si particles, leading to the formation of Si aggregates and clusters. Thick material deposits accumulated on the electrode surface, obstructing the porous channels near the surface and significantly limiting the mass transport and diffusion of electrolytes to the deeper Si particles. Additionally, the cycling process induced several large cracks on electrode surface and cross sections, potentially resulting in the loss of electrical contact and the formation of “dead” Si particles. Collectively, these factors contribute to a shortened cycle life associated with the use of Gen2 electrolytes.

In comparison, the Gen2‐TFEB electrolytes demonstrated a significant decrease in the aggregation of Si particles and a reduction in material deposits on the electrode surface after 50 cycles (Figure 5g–i; S16 and S17, Supporting Information). In the Gen2 electrodes, Si particles predominantly formed aggregates after cycling, resulting in a marked reduction in the number of individual Si particles (Figure 5d and S14, Supporting Information). In contrast, in the Gen2‐TFEB electrodes, Si particles appeared to become spherical and developed a thicker coating or passivation layer, but they remained as individual particles with fewer aggregates forming (Figure 5g and S16, Supporting Information). Besides, the Gen‐TFEB electrodes contained less material deposit on the surface after 50 cycles, enabling the electrodes to maintain a partially porous morphology that facilitates electrolyte penetration and ion transport. These findings explain why the Gen2‐TFEB cells experienced only a 27% capacity loss compared to the 69% capacity loss of Gen2 cells after 50 cycles (Figure 1e).

### 2.5 Calendar Aging

The effects of Li‐free borate additives on the calendar aging of silicon‐based electrodes remain poorly understood due to limited studies. Borate additives can effectively reduce LiPF_6_ salt decomposition and facilitate the formation of polymeric SEI. These features align closely with those of VC, which has demonstrated the potential in slowing down the aging of graphite‐anode lithium‐ion batteries.^[^ ^41^, ^42^ ^]^

**Figure 6:**
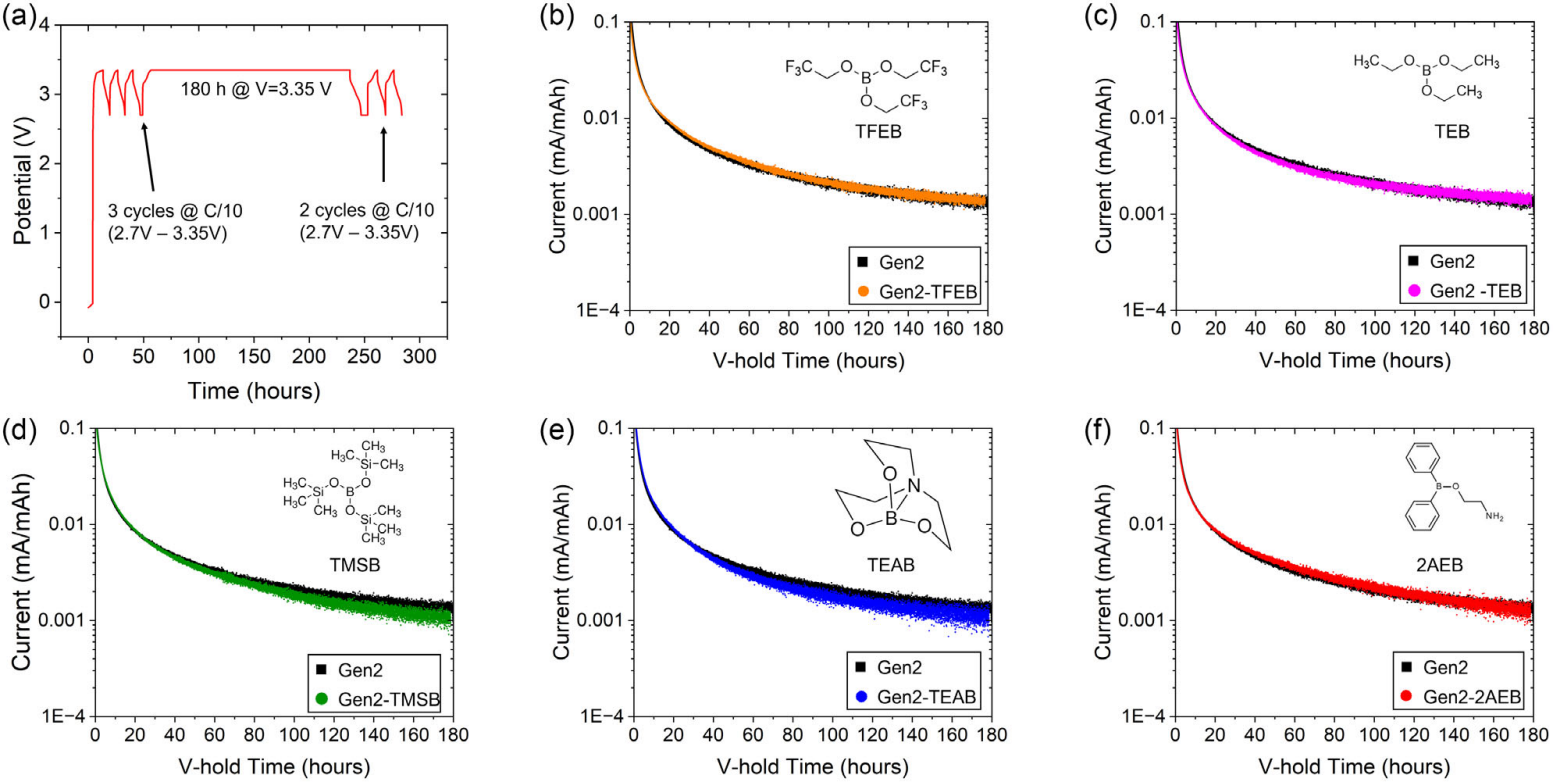
Calendar life performance of LFP||Si full cells with different borate additives. a) Representative calendar aging test procedure developed by the US Department of Energy Silicon Consortium Project. b–f) Average leakage current of LFP||Si full cells over 180 h at 3.35 V for (b) TFEB, (c) TEB, (d) TMSB, (e) TEAB, and (f) 2AEB (sample size, *n* ≥ 3).

To investigate their effect, a calendar aging test process designed by the Department of Energy (DOE) Silicon Consortium Project (SCP) was employed for LFP/Si full cells (**Figure** **6**a). After three formation cycles at a C/10 rate, a 180 h voltage hold (V‐hold) at 3.35 V was performed to measure the leakage current, providing an assessment of the calendar aging process. Subsequently, two diagnostic cycles were carried out at a C/10 rate to evaluate capacity changes. During the early stages of the voltage hold, the leakage current was influenced by both reversible lithiation processes and irreversible electrochemical reactions, including SEI formation and side reactions. As the voltage hold duration exceeded 100 h, the reversible reactions progressively diminished, allowing irreversible reactions to dominate. As shown in Figure 6b–f, the incorporation of 1 v/v% TFEB, 1 v/v% TEB, and 1 wt% 2AEB had a negligible influence on the calendar aging process. In contrast, the addition of 1 v/v% TMSB and 1 wt% TEAB resulted in a slight reduction in leakage current; however, this improvement was not statistically significant. Similarly, for NMC811||Si full cells held at 4.10 V for 180 h, the calendar aging data revealed that the borate additives had a negligible impact on reducing leakage current (Figure S18, Supporting Information). Overall, these findings indicate that the borate additives have limited effectiveness in mitigating leakage current and extending the calendar life of Si‐anode LIBs.

### 2.6 Mechanism

**Figure 7:**
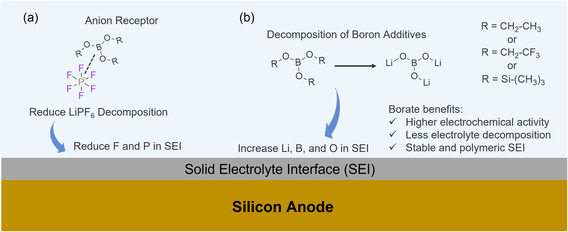
Proposed mechanisms. a) Borate additives serve as anion receptors, interacting with [PF_6_]^−^ anions to reduce their decomposition. b) Borate additives decompose, resulting in the formation of boron‐, lithium‐, and oxygen‐rich SEI layers.

Despite differences in terminal functional groups, borate additives with star‐shaped, three‐branched structures exhibit similar mechanisms for electrolyte stabilization and SEI formation. Boron atoms in borate compounds possess only six valence electrons and exhibit Lewis acid behavior, allowing them to accept a lone pair of electrons to fill the 2p orbital. Thus, in electrolyte systems, they interact with solvents (e.g., ethylene carbonate) or anions (e.g., PF_6_^−^), thereby stabilizing the electrolyte and suppressing its decomposition (**Figure** **7**a). This suppression reduces byproduct formation, minimizing material deposits on electrode surfaces and within pores, which in turn facilitates mass transport, improves material utilization, and prolongs cycle life.

The XPS findings further imply that borate additives play a role in forming an organic‐rich, polymeric SEI, which offers enhanced flexibility to accommodate the volume changes of Si particles. In addition, borate additives exhibit high electrochemical activity, undergoing decomposition to interact with Li^+^ ions (Figure 7b), leading to an SEI enriched in boron, lithium, and oxygen. Collectively, these effects highlight the dual role of borate additives in stabilizing the electrolyte salt and engineering a robust SEI, thereby enhancing electrochemical activity and extending cycle life.

## 3 Conclusion

In this work, all five Li‐free borate additives markedly improved the electrochemical activity and cycling performance of Si‐anode LIBs, indicating their efficacy. Variations in molecular structure and chemical composition led to distinct electrochemical and cycling behaviors, highlighting the critical role of molecular design. A clear positive correlation was observed between electrochemical activity and cycle life, with higher activity yielding superior cycling stability. For instance, TFEB exhibited the highest electrochemical activity and delivered the longest cycle life. In addition, the borate additives decomposed preferentially over the electrolyte salt LiPF_6_, thereby mitigating electrolyte reduction and reducing material deposition on the electrode surface. This preservation of ion transport pathways and electrolyte penetration during cycling contributes to improved overall performance. Furthermore, the borate additives increased Li, O, and B content while reducing F and P content within the SEI. Despite these benefits in cycling performance, their impact on mitigating leakage current and extending the calendar life of Si‐anode LIBs remains limited.

## 4 Experimental Section

The Supporting Information includes detailed descriptions of the materials used and the experimental procedures performed.

## Supporting Information

Supporting Information is available from the Wiley Online Library or from the author.

## Conflict of Interest

The authors declare no conflict of interest.

## Supporting information

Supplementary Material

## Data Availability

The data that support the findings of this study are available from the corresponding author upon reasonable request.
