# Supplementary material for: Impact of Lithium‐Free Borate Additives on the Cycle Life and Calendar Aging of Silicon‐Based Lithium‐Ion Batteries
Source: Small Sci. 2025 Nov 4;6(1):e202500479. doi: 10.1002/smsc.202500479 (PMC12825453; doi:10.1002/smsc.202500479)
Supplement: Supplementary file 1 — Supplementary Material [file SMSC-6-e202500479-s001.pdf]

Supporting Information

Impact of Lithium-free Borate Additives on the Cycle life and Calendar Aging of Silicon-based Lithium-ion Batteries

*Defu Li, Amanda L. Musgrove, Xiuyu Jin, Harry M. Meyer III, Gabriel Muldoon, Gabriel M. Veith, Gao Liu**

Dr. D. Li, Dr. X.Y. Jin, G. Muldoon, Dr. G. Liu

Energy storage and Distributed Resources Division, Lawrence Berkeley National Laboratory, Berkeley, CA, USA.

E-mail: gliu@lbl.gov

Dr. A.L. Musgrove, Dr. H.M. Meyer, Dr. G.M. Veith,

Chemical Sciences Division, Oak Ridge National Laboratory, Oak Ridge, TN, USA

Experimental methods:

Materials:

Tris(2,2,2-trifluoroethyl) borate (TFEB), triethyl borate (TEB), tris(trimethylsilyl) borate (TMSB), 2-aminoethyldiphenyl borate (2AEB), and triethanolamine borate (TEAB) were purchased from Sigma-Aldrich and were used directly without additional treatment. The Gen2 electrolytes were obtained from Argonne National Laboratory and comprised 1.2 M $LiPF_6$ dissolved in a liquid mixture of ethylene carbonate (EC) and ethyl methyl carbonate (EMC) with a volume ratio of 3:7. All NMC811, LFP, and silicon-based electrodes were prepared at the Cell Analysis, Modeling, and Prototyping (CAMP) facility at Argonne National Laboratory. The NMC811 cathode (2.59 mAh/cm^2) contained 90 wt% $LiNi_{0.8}Mn_{0.1}Co_{0.1}O_2$, 5 wt% C45 carbon (Timcal), and 5 wt% PVDF binder (Solvay 5130). The silicon-based electrodes contained 80 wt% silicon (Oak), 10 wt% C45 carbon, and 10 wt% PF84 polyimide, with areal capacities of 1 mAh/cm^2 or 2.0 mAh/cm^2. The Si electrodes with 1 mAh/cm^2 were used for calendar aging tests, whereas those with 2 mAh/cm^2 were employed for cycle life tests. The LFP cathode contained 90 wt% $LiFePO_4$ (Johnson Matthey), 5 wt% C45 carbon (Timcal), and 5 wt% PVDF binder (Solvay 5130), with an areal capacity of 2.66 mAh/cm^2.

Electrolyte Preparation:

TFEB, TEB, and TMSB are liquid at room temperature, whereas 2AEB and TEAB are solid. Thus, when preparing targeted electrolytes by mixing Gen2 electrolyte and borate additives, a volume-by-volume method was employed for TFEB, TEB, and TMSB, while a weight-by-weight method was used for 2AEB and TEAB. All electrolytes were prepared inside a glovebox filled with argon gas.

Battery Assembly and Cycle Life Test

All half-cell and full-cell batteries were assembled using Hohsen coin cells (2032) within an argon-filled glovebox. The silicon-based anode electrodes were punched to a diameter of 9/16", while NMC811 and LFP cathodes were cut to a diameter of 1/2". In half-cell studies, lithium chips (16 mm in diameter, MTI Co.) were used as the anode, and the silicon-based electrodes served as the cathode. In full-cell studies, the silicon-based electrodes were utilized as the anode, while the NMC811 and LFP electrodes were used as the cathodes. Each coin cell was filled with 120 μL of electrolytes, and Celgard 2400 separators (19 mm in diameter) were employed to isolate the positive and negative electrodes. The NMC811 cathodes and silicon-based anodes were utilized to assemble full-cell batteries for cycle life testing. The initial three cycles were conducted at C/20 to facilitate SEI formation, followed by subsequent cycles performed at a C/3 rate.

Calendar Life Test

Calendar aging tests were performed on full-cell batteries assembled with silicon-based anode (areal capacity ≈ 1 mAh/cm^2) and the LFP cathodes (areal capacity ≈ 2.66 mAh/cm^2). The larger areal capacity of LFP cathode was designed to provide sufficient Li^+ inventory during the hold-voltage period. The calendar aging test followed the Tier 2 protocol designed by the U.S. Department of Energy Silicon Consortium Project (SCP). First, the cells were rested at open-circuit voltage (OCV) rest for 4 hours. Subsequently, three formation cycles were performed at a C/10 rate within a voltage range of 2.7 V to 3.35 V, followed by holding the cells at 2.7 V until the current decreased below C/100 to fully delithiate the silicon-based electrodes. Then, the cells were charged to 3.35 V and held at 3.35 V for 180 hours, after which they were discharged to 2.7 V and held at 2.7 V to fully delithiate the electrodes. Finally, two diagnostic cycles were performed at a C/10 rate. A similar protocol was applied

to NMC811 cathode (areal capacity ≈ 2.6 mAh/cm^2), but using a voltage window of 3.0-4.10 V, with the cell held at 4.10 V for 180 hours.

Electrochemical Characterization

For cyclic voltammetry characterization, coin cells comprising lithium metal, a Celgard 2400 separator, and Si-based negative electrodes were assembled and tested at 30 °C. The cells were rested at OCV for 4 hours, and then discharged with the linear sweep voltammetry (LSV) method from OCV to 0 V. Subsequently, the cells were cycled between 0V and 3V for 20 cycles at a rate of 0.1 mV/s. Impedance measurements were carried out on coin cells assembled with an NMC811 cathode, a Celgard 2400 separator, and silicon-based negative electrodes. Measurements were recorded before and after three cycles of SEI formation.

Ionic conductivity experiments were performed on coin cells assembled with two spacers (16 mm diameter) and a Celgard 2400 separator (25 μm thickness). An excess volume of electrolyte was used to ensure complete immersion of the separator. After allowing the cells to reach a steady state at OCV for 12 hours, EIS experiments were performed. In a Nyquist plot format, all resulting data appeared almost linear, straight lines. The interception between the line and X-axis was treated as electrolytes resistance (R). The ionic conductivity (σ) of the electrolytes is calculated using the formula $\sigma = l/(R \cdot A)$. In this experimental setup, l represents the thickness of separator, A denotes the spacer surface area.

XPS Experiment:

XPS experiments were performed using a Thermo Scientific Model Nexsa G2 XPS instrument to examine the surface composition and bonding properties of the SEI surface. For sample preparation, coin cells were assembled with the Si negative electrodes, lithium metal, and various electrolytes. Following three cycles of SEI formation cycles, coin cells were disassembled, and the Si negative electrodes were washed with dimethyl carbonate (DMC) solvent within a glovebox. The samples were transferred to the XPS instrument using a vacuum transfer module to avoid air exposure. This instrument utilizes monochromatic, micro-focused, Al K_α X-rays (1486.6 eV) for measurement and data acquisition. A 400 µm X-ray spot size was selected to maximize signal intensity and offer an average surface composition over the maximum possible surface area. Surrey spectra were acquired at a pass energy of 200 eV for qualitative and quantitative analysis, while high-resolution core-level spectra were collected at a pass energy of 50 eV to enable detailed chemical state analysis.

FIB-SEM Experiments

The coin cells (2032, Hohsen Co.) were assembled inside an argon-filled glovebox using the NMC811 cathodes (2.59 mAh/cm^2) and Si-based electrodes (2.0 mAh/cm^2). A Celgard 2400 separator were placed between the positive and negative electrodes. Each cell was injected with a total of 120 µL electrolytes. The full cells underwent cycling for a total of 50 cycles at a rate of 1/3C. The cycling process was terminated at the discharged state to ensure that the Si electrodes were in a delithiated condition. The coin cells were disassembled using Hohsen disassembly tool. The Si electrodes were then prepared for focused ion beam- scanning electron microscope (FIB-SEM) experiments without washing. All preparations were conducted within a glovebox to minimize air exposure. The FIB-SEM experiments were conducted at the National Center for Electron Microscopy (NCEM), Laurence Berkeley National Laboratory. The Si electrodes were initially ion milled using a high current (9 nA, 30 kV), followed by milling at a lower current (0.4 nA, 30 kV) for polishing.

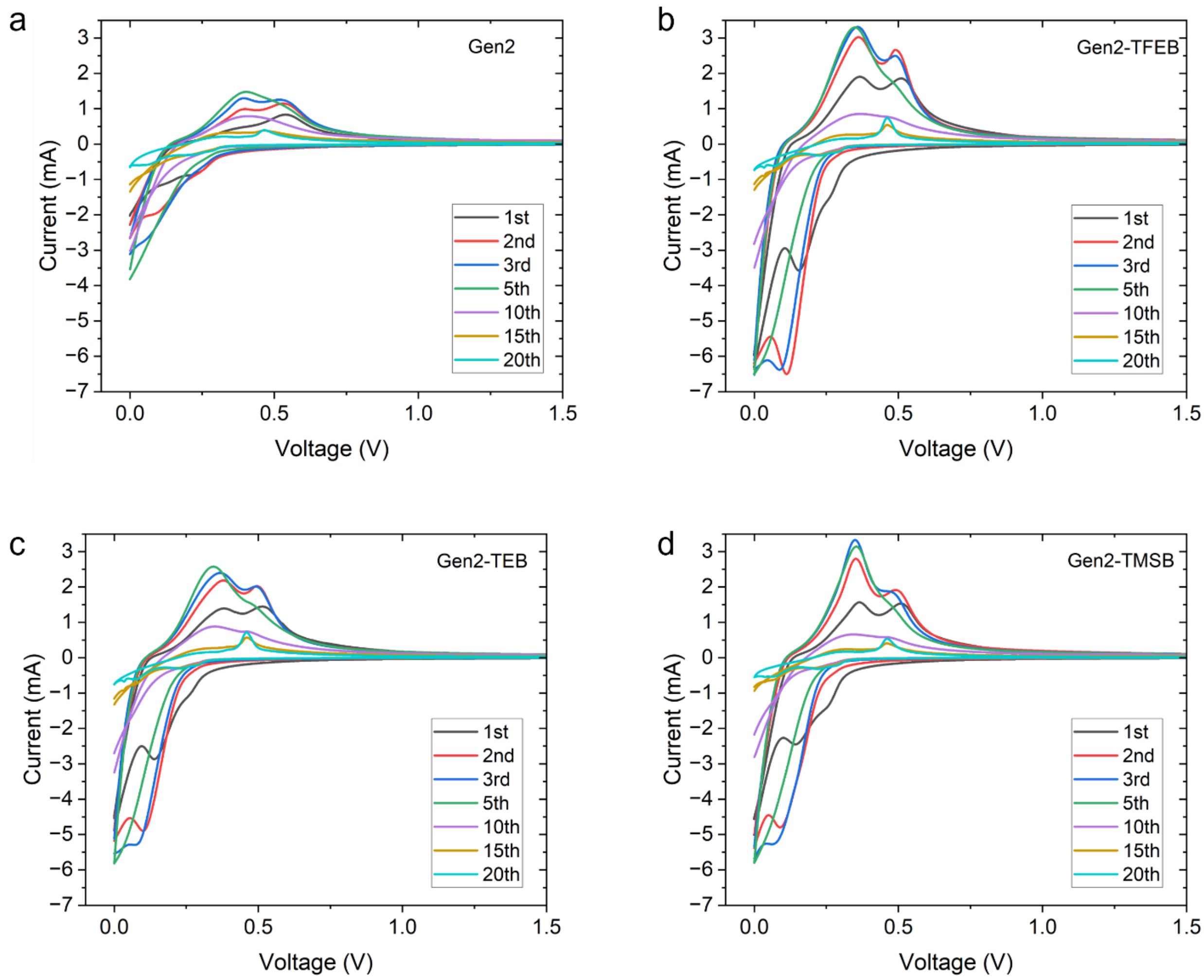

Figure S1. Cyclic voltammetry results of Li||Si cells with different electrolyte formulations at different cycle numbers. (A) Gen2, (B) Gen2-TFEB, (C) Gen2-TEB, (D) Gen2-TMSB.

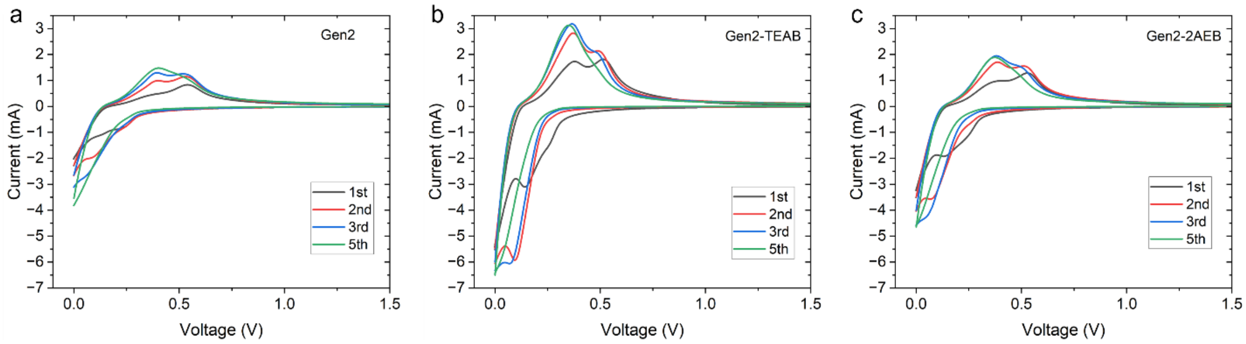

Figure S2. Cyclic voltammetry results of Li||Si cells with different electrolyte formulations at different cycle numbers. (A) Gen2, (B) Gen2-TEAB, (C) Gen2-2AEB.

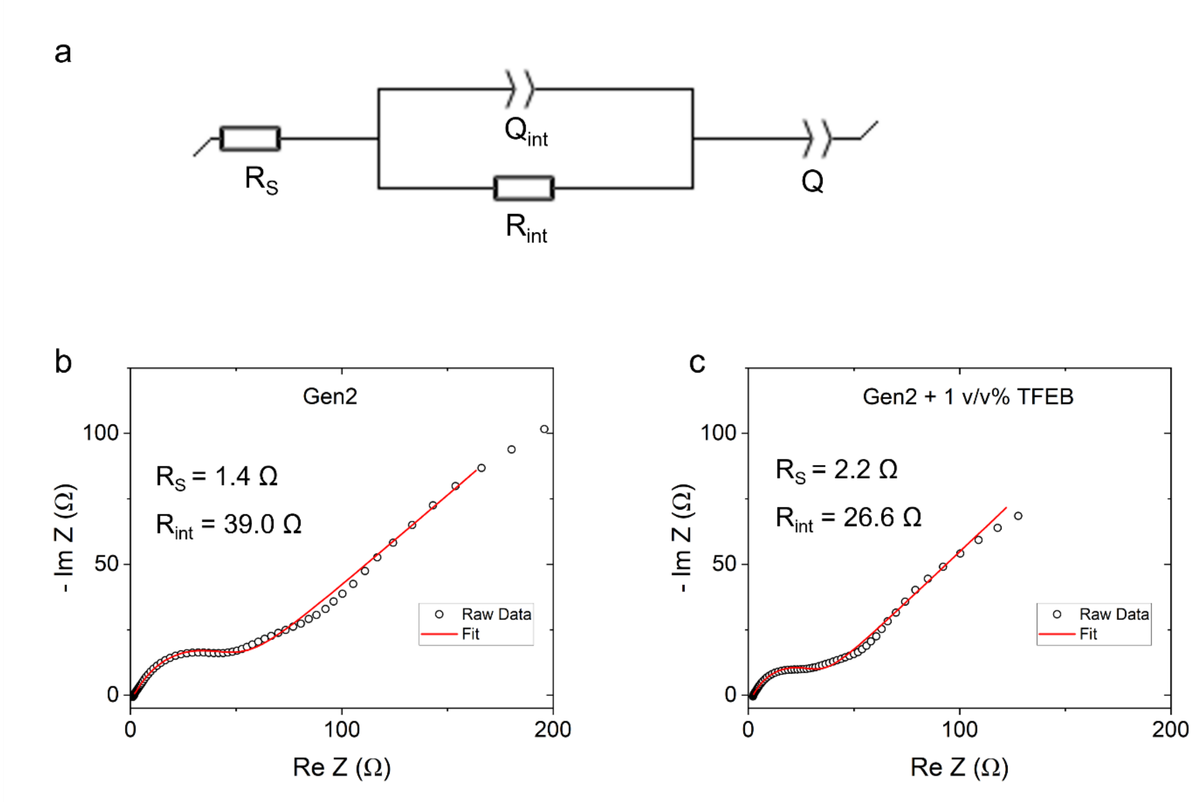

Figure S3. (a) Equivalent circuit. The R_s refers to the ohmic resistance, and R_{int} refers to the interfacial resistance. (b, c) Nyquist plots for the cells using (b) Gen2 and (c) Gen2-TFEB electrolytes.

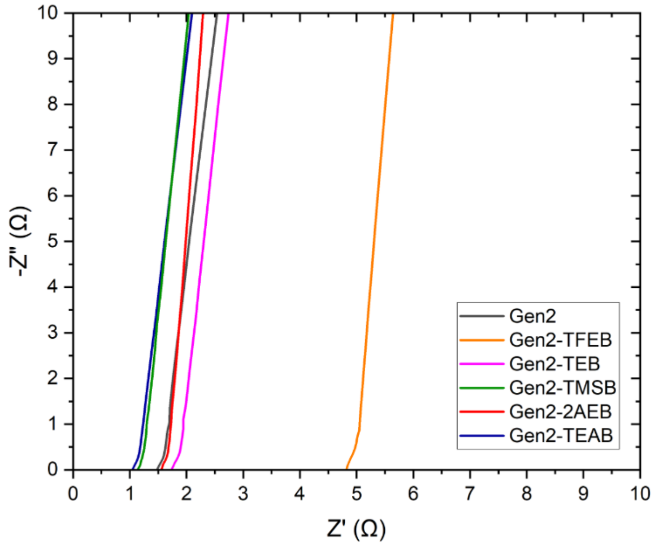

Figure S4. Representative electrochemical impedance spectra of the Gen2 electrolytes and Gen2 electrolytes with borate additives.

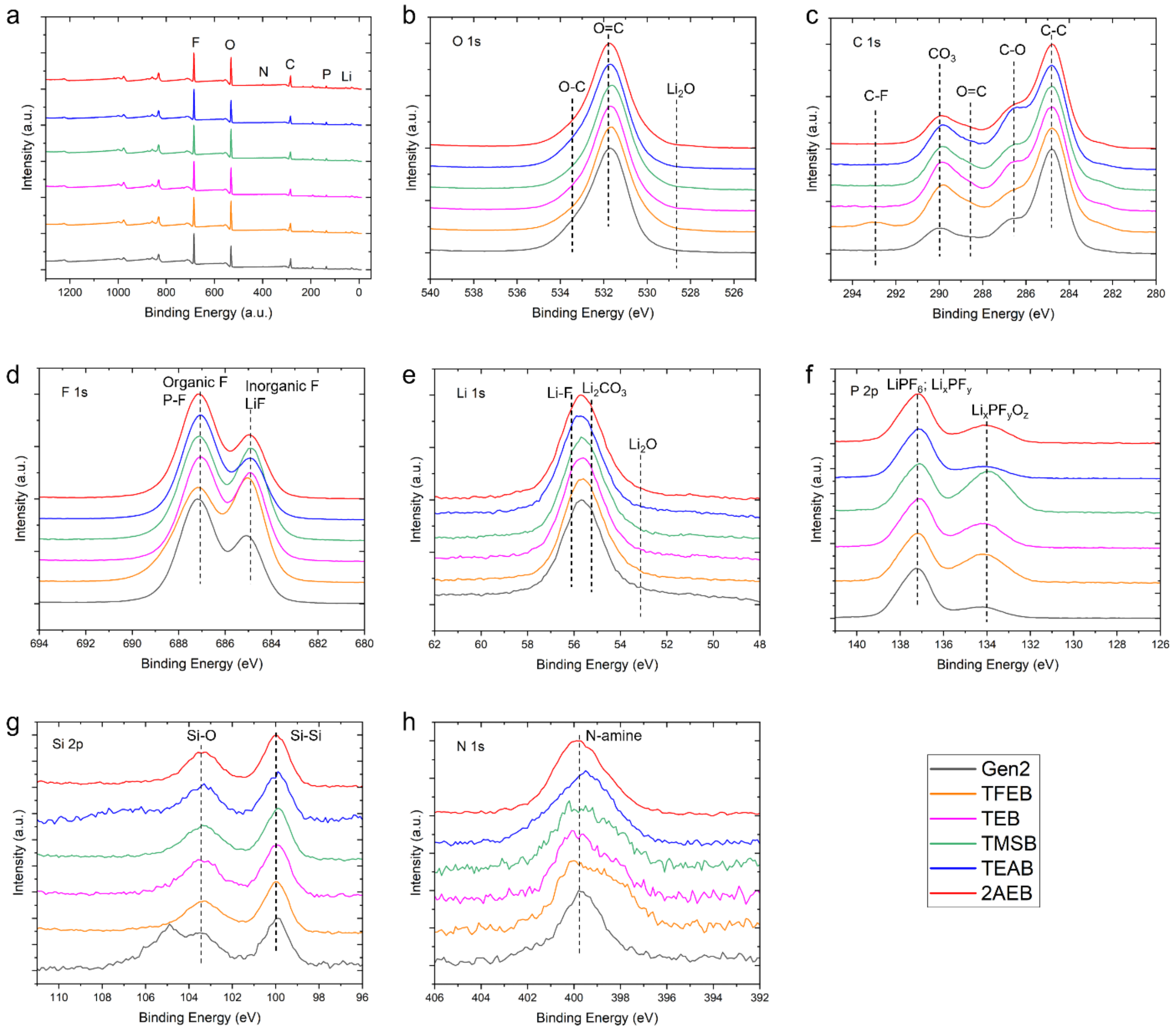

Figure S5. Summarized XPS spectra of (a) all major elements, (b) oxygen, (c) carbon, (d) fluorine, (e) lithium, (f) phosphorus, (g) silicon, and (h) nitrogen.

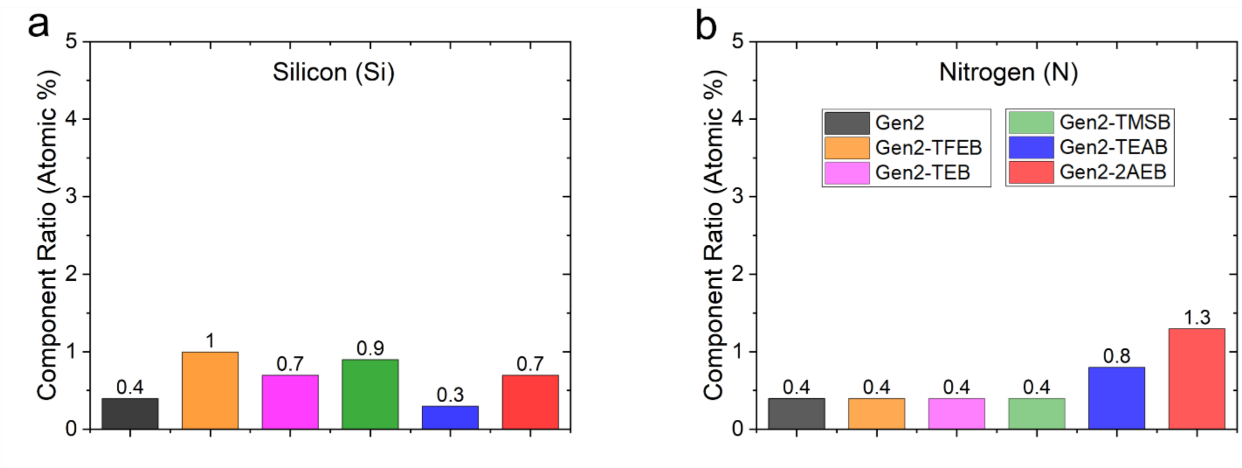

Figure S6. SEI surface composition ratio of (a) Silicon, (b) nitrogen.

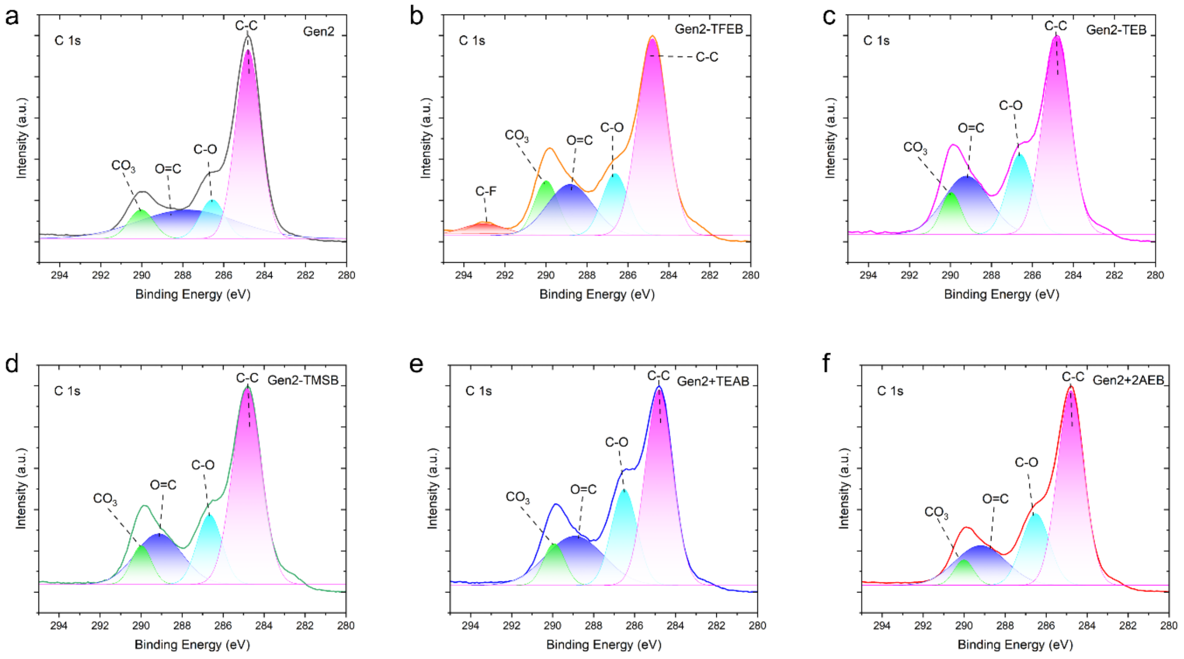

Figure S7. XPS C 1s spectra with peak-fitting profiles for (a) Gen2, (b) Gen2-TFEB, (c) Gen2-TEB, (d) Gen2-TMSB, (e) Gen2-TEAB, and (f) Gen2-2AEB.

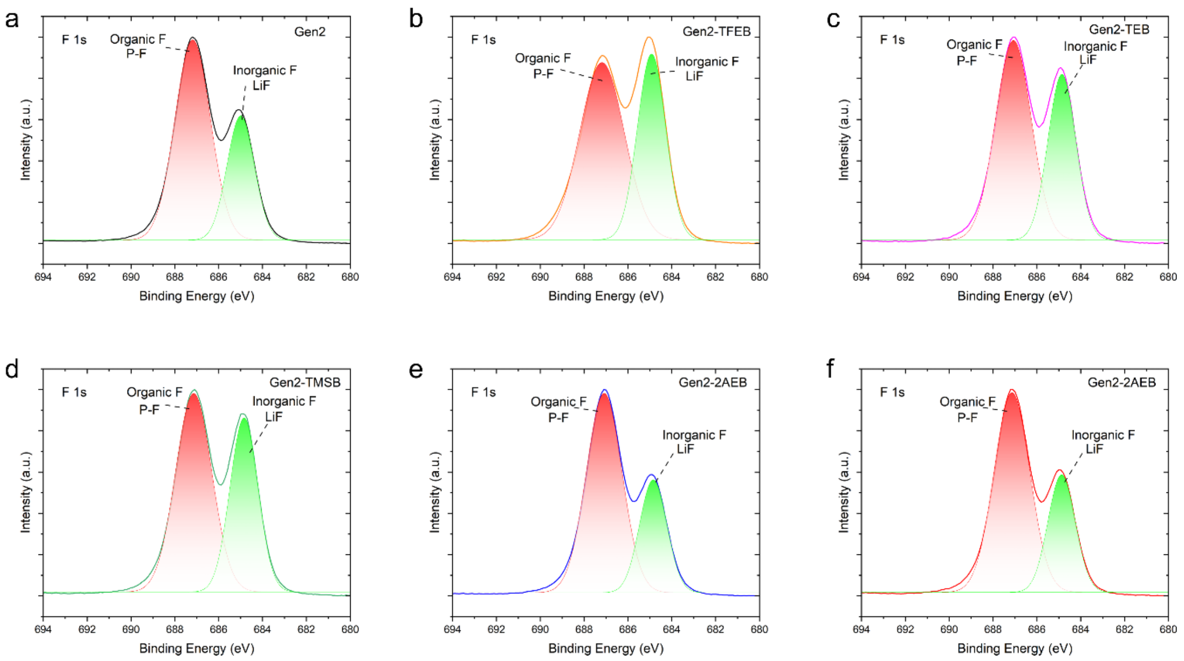

Figure S8. XPS F 1s spectra with peak-fitting profiles for (a) Gen2, (b) Gen2-TFEB, (c) Gen2-TEB, (d) Gen2-TMSB, (e) Gen2-TEAB, and (f) Gen2-2AEB.

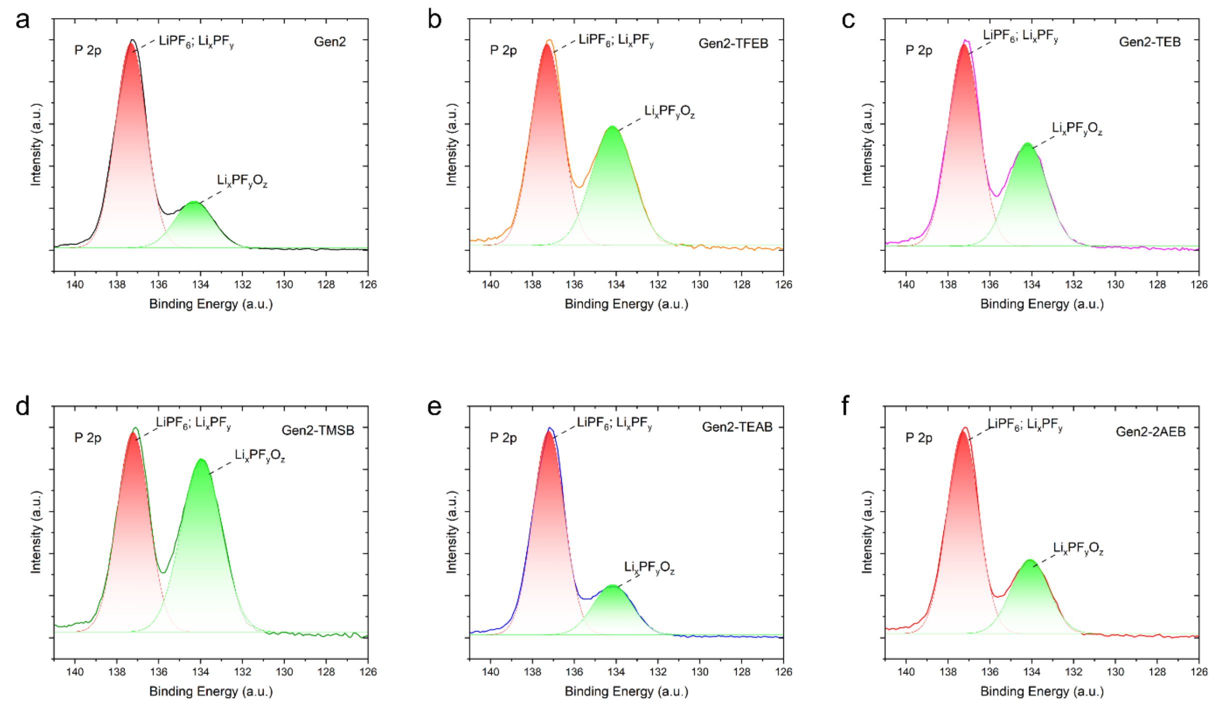

Figure S9. XPS P 2p spectra with peak-fitting profiles for (a) Gen2, (b) Gen2-TFEB, (c) Gen2-TEB, (d) Gen2-TMSB, (e) Gen2-TEAB, and (f) Gen2-2AEB.

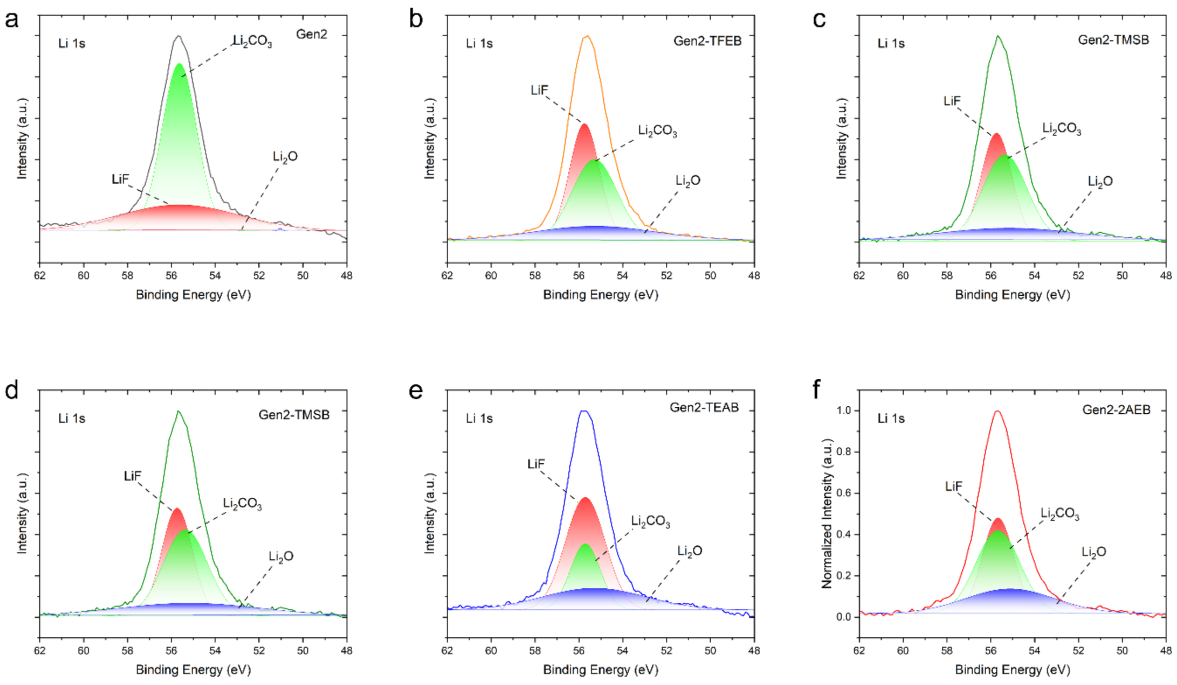

Figure S10. XPS Li 1s spectra with peak-fitting profiles for (a) Gen2, (b) Gen2-TFEB, (c) Gen2-TEB, (d) Gen2-TMSB, (e) Gen2-TEAB, and (f) Gen2-2AEB.

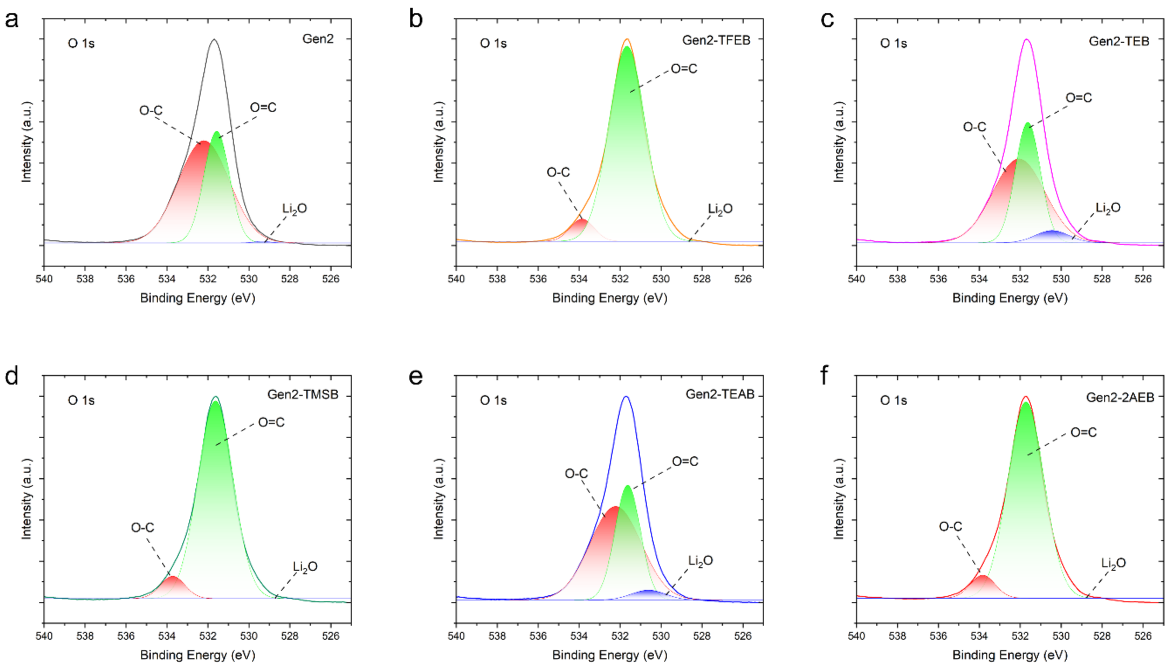

Figure S11. XPS O 1s spectra with peak-fitting profiles for (a) Gen2, (b) Gen2-TFEB, (c) Gen2-TEB, (d) Gen2-TMSB, (e) Gen2-TEAB, and (f) Gen2-2AEB.

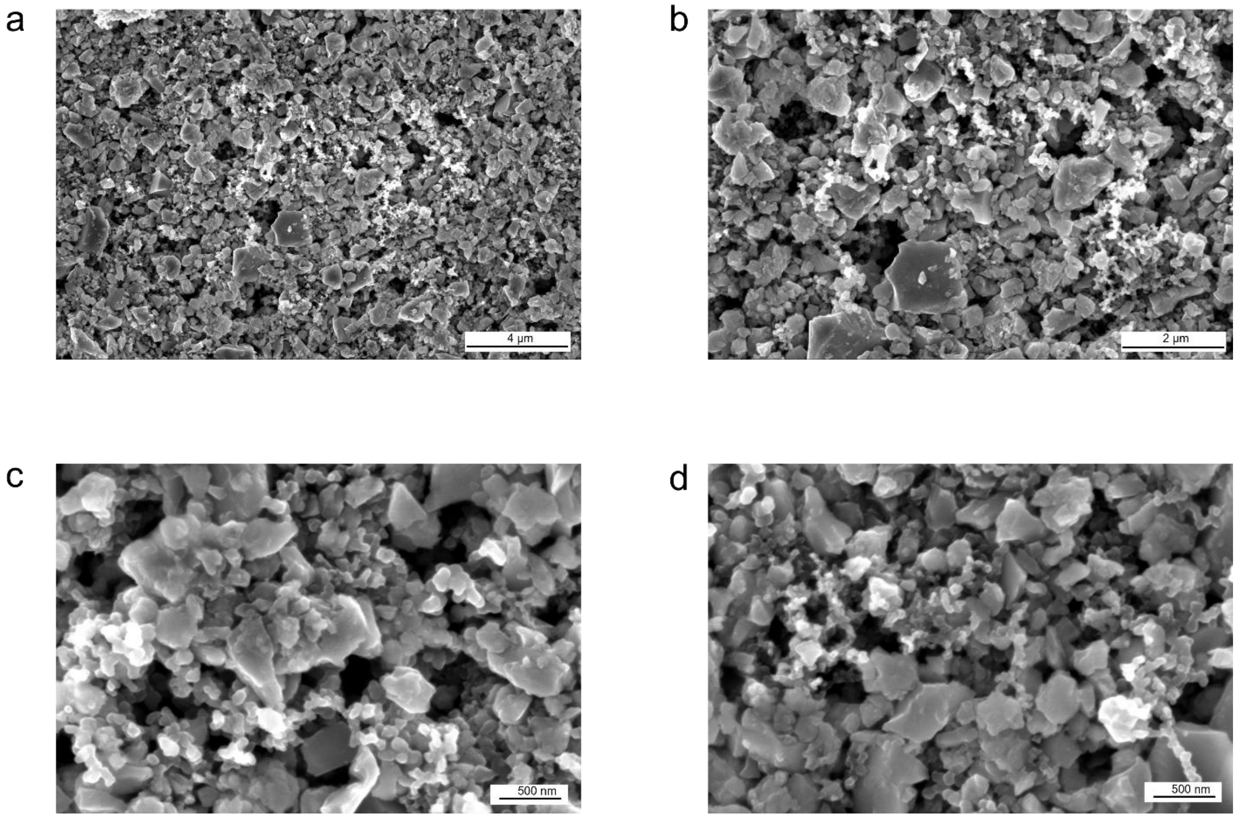

Figure S12. Surface SEM images of pristine Si electrodes.

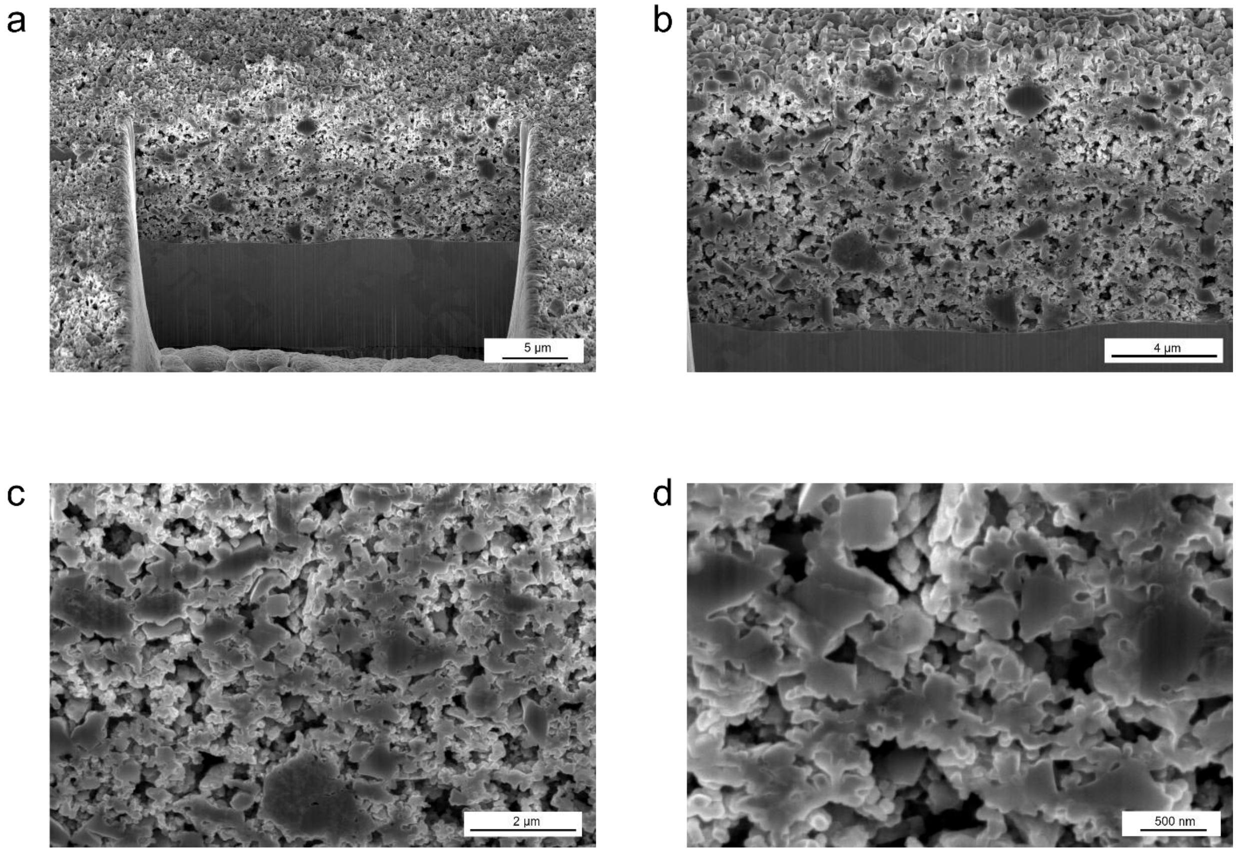

Figure S13. FIB-SEM cross-sectional morphology of pristine Si electrodes.

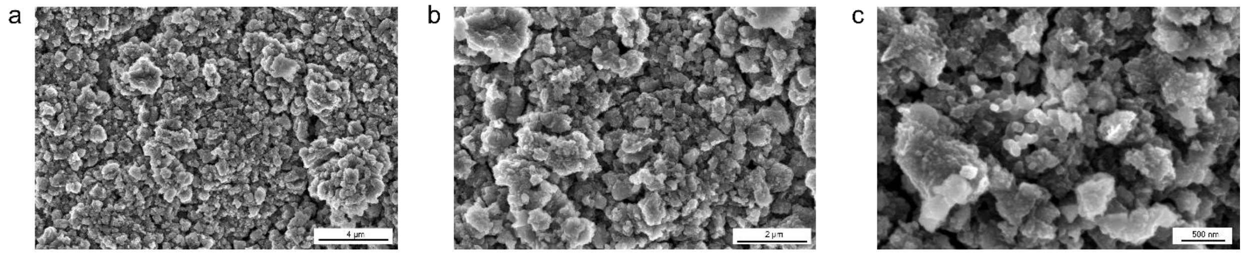

Figure S14. Surface SEM of Si electrode after 50 cycles using Gen2 electrolytes.

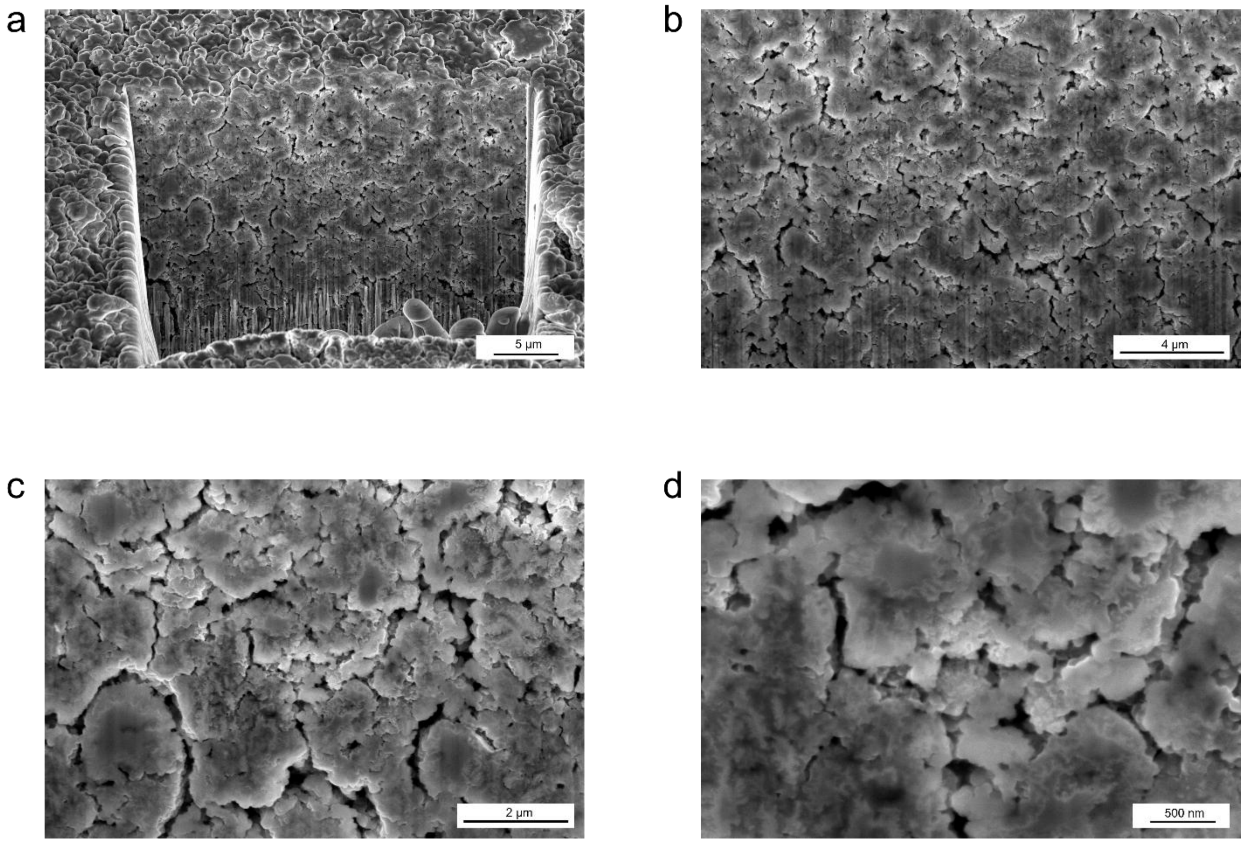

Figure S15. FIB-SEM cross-sectional morphology of pristine Si electrodes after 50 cycles using Gen2 electrolytes.

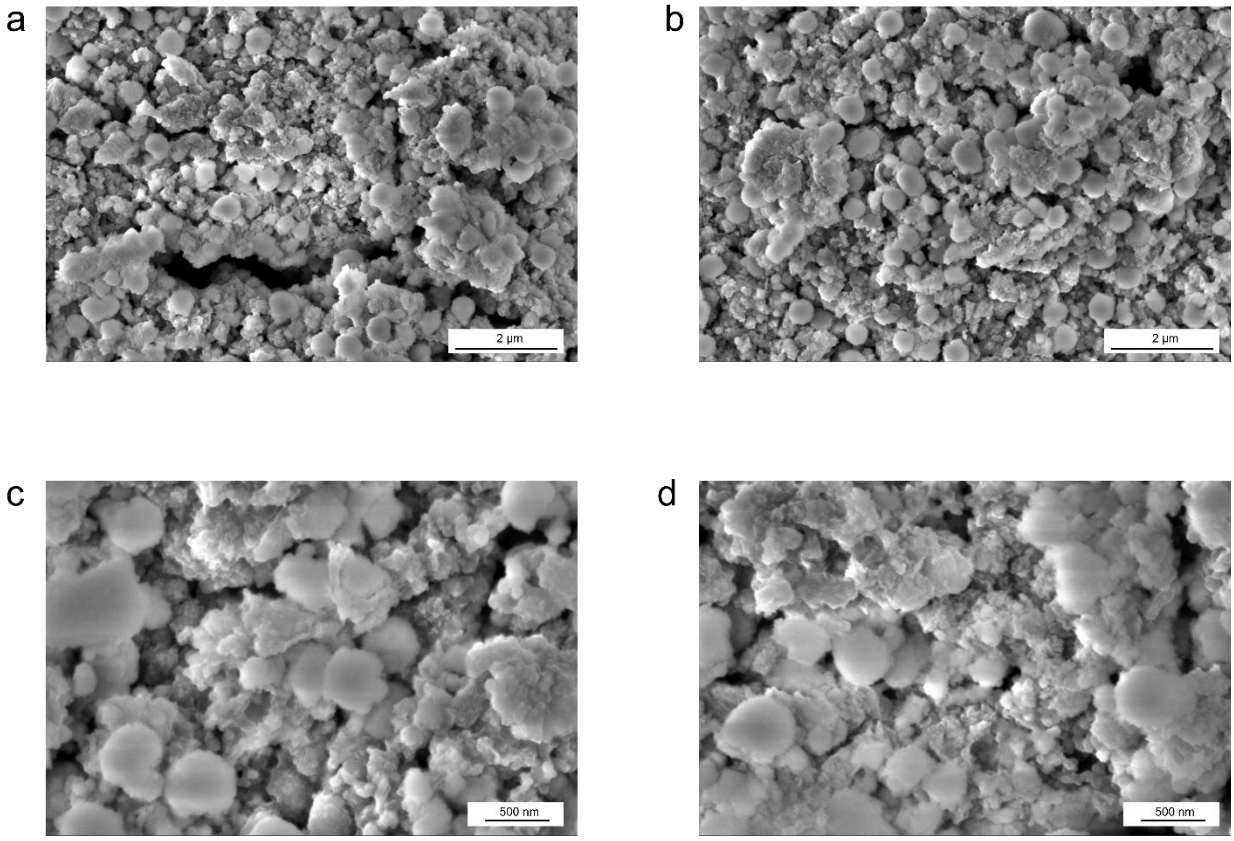

Figure S16. Surface SEM of Si electrode after 50 cycles using Gen2-TFEB electrolytes.

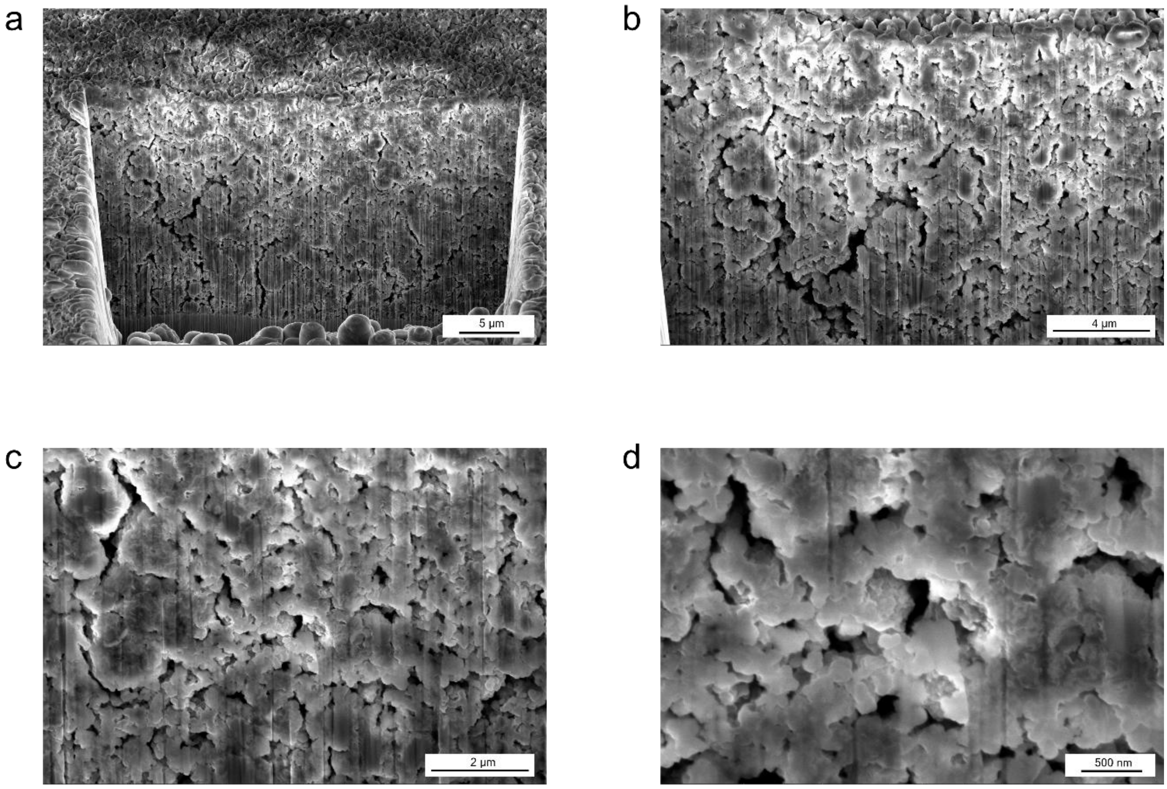

Figure S17. FIB-SEM cross-sectional morphology of pristine Si electrodes after 50 cycles using Gen2-TFEB electrolytes.

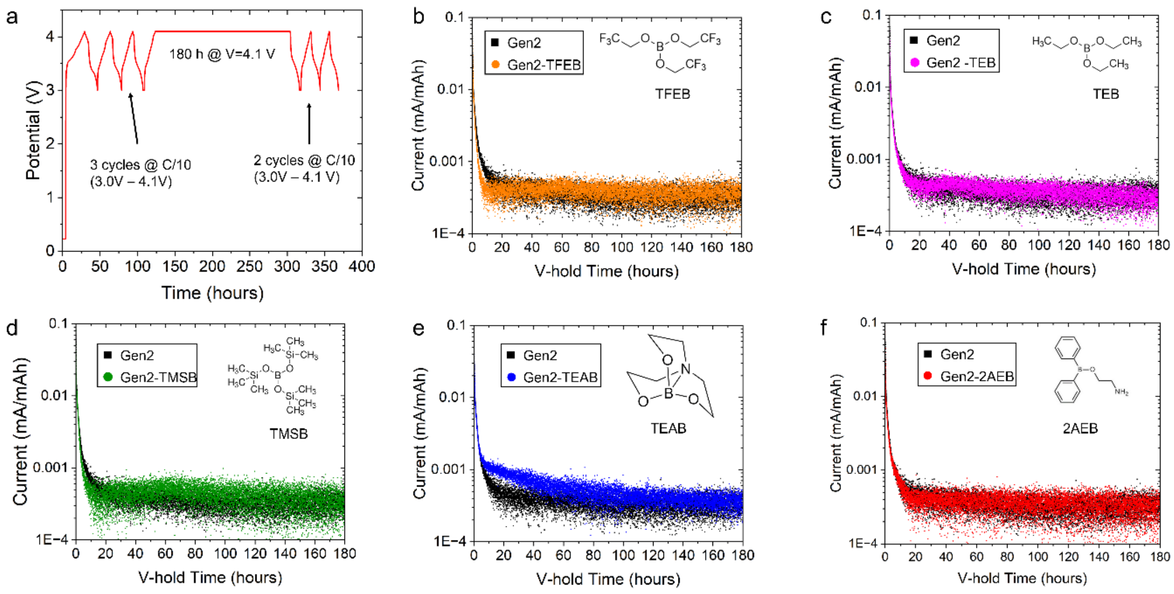

Figure S18. Representative calendar life performance of NMC811||Si full cells with different borate additives. (a) Representative calendar aging test procedure. (b-f) Average leakage current of NMC811||Si full cells over 180 hours at 4.1 V for (b) TFEB, (c) TEB, (d) TMSB, (e) TEAB, and (f) 2AEB.
